# Long-term Effects of a Social Media–Based Intervention (Run4Love) on Depressive Symptoms of People Living With HIV: 3-Year Follow-up of a Randomized Controlled Trial

**DOI:** 10.2196/36809

**Published:** 2022-06-28

**Authors:** Yan Guo, Yingqi Li, Chuanchuan Yu, He Xu, Y Alicia Hong, Xiaolan Wang, Nanxiang Zhang, Yu Zeng, Aliza Monroe-Wise, Linghua Li, Cong Liu, Weiping Cai, Aihua Lin

**Affiliations:** 1 Department of Medical Statistics School of Public Health Sun Yat-sen University Guangzhou China; 2 Department of Population and Quantitative Health Sciences University of Massachusetts Chan Medical School Worcester, MA United States; 3 Department of Health Administration and Policy, College of Health and Human Services George Mason University Fairfax, VA United States; 4 Department of Global Health University of Washington Seattle, WA United States; 5 Department of Infectious Diseases Guangzhou Eighth People’s Hospital Guangzhou China; 6 Department of Health Service and Management Guangzhou Xinhua University Guangzhou China

**Keywords:** HIV, depressive symptoms, mobile health, mHealth, social media–based, long-term intervention effect

## Abstract

**Background:**

Emerging studies have shown the effectiveness of mobile health (mHealth) interventions in reducing depressive symptoms among people living with HIV. Most of these studies included only short-term follow-up, with limited data on long-term effects.

**Objective:**

The purpose of this study is to assess the long-term effects of a randomized controlled trial called Run4Love on depressive symptoms among people living with HIV at 1-year and 3-year follow-ups.

**Methods:**

A total of 300 people living with HIV with depressive symptoms were recruited and randomized to an intervention or a control group in Guangzhou, China, from September 2017 to January 2018. The intervention group received a 3-month Run4Love program, including adapted evidence-based cognitive behavioral stress management courses and exercise promotion via WeChat (Tencent), a popular social media app. The control group received usual care and a brochure on nutrition. The primary outcome was reduction in depressive symptoms, measured using the Center for Epidemiological Studies–Depression (CES-D) scale. Data used in this study were collected at baseline and at the 1-year and 3-year follow-ups. Generalized estimating equations were used to examine the group differences at 1-year and 3-year follow-ups.

**Results:**

Approximately half of the participants completed the assessment at 1-year (149/300, 49.7%) and 3-year (177/300, 59%) follow-ups. At 1-year follow-up, participants in the intervention group reported significant reduction in depressive symptoms compared with the control group (CES-D: from 23.9 to 18.1 in the intervention group vs from 24.3 to 23.3 in the control group; mean −4.79, SD 13.56; 95% CI −7.78 to −1.81; *P*=.002). At 3-year follow-up, between-group difference in CES-D remained statistically significant (from 23.9 to 20.5 in the intervention group vs from 24.3 to 24.4 in the control group; mean −3.63, SD 13.35; 95% CI −6.71 to −0.54; *P*=.02). No adverse events were reported during the 3-year follow-up period.

**Conclusions:**

The mHealth intervention, Run4Love, significantly reduced depressive symptoms among people living with HIV, and the intervention effects were sustained at 1-year and 3-year follow-ups. Further research is needed to explore the mechanisms of the long-term effects of mHealth interventions such as Run4Love and to implement these effective interventions among people living with HIV.

**Trial Registration:**

Chinese Clinical Trial Registry ChiCTR-IPR-17012606; https://trialsearch.who.int/Trial2.aspx?TrialID=ChiCTR-IPR-17012606

**International Registered Report Identifier (IRRID):**

RR2-10.2196/10274

## Introduction

More than one-third of people living with HIV experience depressive symptoms, which present a major health concern and are associated with other negative health outcomes such as poor adherence to antiretroviral therapy and increased morbidity and mortality [1-5]. In addition, people living with HIV with depressive symptoms are more likely to engage in HIV-related risk behaviors such as inconsistent use of condoms and substance abuse, which increases the risk of HIV infection and transmission [1,6,7]. As depression is often chronic and requires long-term follow-up, effective interventions to address this common comorbidity in people living with HIV with long-term effects are urgently needed [7].

However, mental health resources are scarce, especially in low-income and middle-income countries, and <17% of people living in these countries have received the needed mental health treatment [8,9]. For example, there is 0.5 psychiatrist per million people in Africa, which has the highest burden of HIV, and 22 psychiatrists per million people in China, compared with 83 psychiatrists per million people in Europe, 105 psychiatrists per million people in the United States, and 147 psychiatrists per million people in Canada [10,11]. Therefore, interventions to provide effective treatments for depression and facilitate the implementation of such treatments for people living with HIV living in low-income and middle-income countries are urgently needed.

A growing number of mobile health (mHealth) interventions have been developed to improve mental health outcomes, especially depressive symptoms, in people living with HIV. However, the existing literature has several limitations. First, few studies have explored the long-term effects of mHealth interventions [12-14]. A recent systematic review and meta-analysis included 14 randomized controlled trials (RCTs) of mHealth interventions for reducing depressive symptoms among people living with HIV [12]. Among these, only one study explored the long-term intervention effects for up to 1 year, and the results did not show any improvement in depressive symptoms [15]. Second, most mHealth studies have small sample sizes <100; only a few studies have sample sizes >200 [15-18]. Third, existing mHealth interventions among people living with HIV have largely been conducted in high-income countries such as the United States and Western Europe, with only a few studies conducted in low-income or middle-income countries [15,16,19-21]. Finally, existing mHealth interventions are mostly telephone-based or simply send SMS text messages, whereas few studies have used web applications or mobile apps [12,15,16,19,22-24].

Our mHealth intervention, Run4Love, is a 3-month internet-based multimedia program delivered via a popular social media app called WeChat. Run4Love was effective in reducing depressive symptoms in people living with HIV at 3-, 6-, and 9-month follow-ups in a 2-arm parallel RCT with a sample size of 300 [16]. After the trial, we continued to follow all the participants for 3 years after the baseline. In this study, we aimed to evaluate the long-term effects of the Run4Love intervention at 1-year and 3-year follow-ups. We hypothesized that the Run4Love intervention had long-term intervention effects in reducing depressive symptoms at 1-year and 3-year follow-ups and that it also had long-term effects on secondary outcomes such as the patients’ quality of life (QOL), positive coping, and perceived stress.

## Methods

### Overview

Participants were recruited from the outpatient department of Guangzhou Eighth People’s Hospital, which is the largest infectious disease hospital in Guangzhou, the capital city of Guangdong Province in South China, from September 2017 to January 2018. Participants were randomized to the Run4Love intervention group or the control group in 1:1 ratio.

### Ethics Approval

The Run4Love intervention was registered in the Chinese Clinical Trial Registry (ChiCTR-IPR-17012606). The study design is described in the CONSORT-EHEALTH (Consolidated Standards of Reporting Trials of Electronic and Mobile Health Applications and Online Telehealth) checklist in Multimedia Appendix 1. The study protocol was approved by the institutional review board of the School of Public Health at the Sun Yat-sen University (approval number 2015-28).

### Participants

Participants were screened for depressive symptoms, and those with depressive symptoms were invited to participate in the study. A total of 300 participants were recruited for the study. Inclusion criteria were the following: (1) aged ≥18 years, (2) being HIV seropositive, (3) having depressive symptoms (Center for Epidemiological Studies–Depression [CES-D] scale score ≥16), (4) willing to provide hair samples, and (5) using WeChat. Exclusion criteria were the following: (1) unable to complete the screening or baseline questionnaire, (2) having trouble in reading or listening to the intervention materials, (3) currently on treatment for depressive symptoms, and (4) unable to participate in physical activities. All the recruited participants signed the informed consent form. Study participants were given up to 10 Yuan (approximately US $1.6) as weekly incentive for their completion of the Run4Love program; they were also provided 20 to 50 Yuan (US $3.1-US $7.8) for completion of the follow-up questionnaires.

### Procedure

#### Run4Love RCT

After completing the baseline assessment, eligible patients were randomly assigned to the Run4Love intervention group or the wait-list control group. Randomization was performed using SAS software (version 9.4) to generate a randomization list with a block size of 4. Owing to the design of the study, neither the participants nor the researchers were blinded. The Run4Love intervention protocol has been described elsewhere [25]. Briefly, the 3-month Run4Love intervention program was tailored to people living with HIV with depressive symptoms. The intervention included adapted cognitive behavioral stress management (CBSM) courses and physical activity promotion. The adapted CBSM courses consisted of 12 weekly sessions on stress management and coping skills. Physical activity promotion provided guidance and suggestions for regular exercise and healthy diet choices.

Personalized feedback based on completion of the CBSM courses was sent to each participant on a weekly basis via a novel, enhanced WeChat platform developed by the investigators. Corresponding financial incentives were also sent based on their completion status on a weekly basis. In addition, participants in the intervention group received 5 phone calls from the research staff at 1 week and 1, 2, 5, and 8 months after enrollment. The purposes of these phone calls were to confirm, facilitate, and sustain the participation of the patients.

Participants in the wait-list control group received a paper-based brochure on nutrition for people living with HIV at baseline, and they received the Run4Love program at 9 months from baseline, delivered in the same way as in the intervention group for 3 months, with the same materials and frequencies, but without any phone calls and incentives.

#### Data Collection Procedure

Participants in both groups were invited to complete in-person assessments at 3, 6, and 9 months and web-based assessments at 1 and 3 years after enrollment via the enhanced WeChat platform. Measurements in the follow-up questionnaires were the same across time and took approximately 15 to 25 minutes to complete. Except for the 9-month assessment, participants received a compensation of 20 Yuan (approximately US $3) for completing the survey. Reminders were sent and a follow-up call was made to those who were not responsive for a week.

### Measures

#### Primary Outcome

The primary outcome was reduction in depressive symptoms, which was measured by the Chinese version of the 20-item CES-D scale at baseline and at 3-, 6-, 9-, 12-, and 36-month follow-ups. Changes in CES-D score at each time point from that at baseline were calculated [26,27]. The CES-D scale is a validated measurement that is used among various populations, including Chinese people living with HIV with depressive symptoms [27-29]. The internal consistency of the scale was satisfactory, with most Cronbach α being >.80 when measured at all time points. Items of the scale were assessed on a 4-point Likert scale for the frequency of depressive behaviors or feelings in the past week, such as “I was bothered by things that usually don’t bother me.” The total score ranges from 0 to 60, with score ≥16 indicating depressive symptoms.

#### Secondary Outcomes

Secondary outcomes included QOL, perceived stress, positive and negative coping, self-efficacy, HIV-related stigma, and depression severity.

QOL was assessed using the 31-item World Health Organization Quality of Life HIV short version (WHOQOL-HIV BREF) [30,31]. Items were assessed on a 5-point Likert scale for QOL in the past 2 weeks, such as “How much do you enjoy life?” with total score ranging from 24 to 120. Perceived stress was measured using the 10-item Perceived Stress Scale (PSS), where the total score ranges between 0 and 40, with high score indicating more stress in the past month [32]. Coping was measured using the Simplified Ways of Coping Questionnaire (SWCQ), which includes a 12-item positive coping subscale (score range 0-36) and an 8-item negative coping subscale (score range 0-24) [33]. Self-efficacy was assessed using the 10-item Chinese version of the General Self-Efficacy Scale. The total score ranges between 10 and 40, with high score indicating better self-efficacy [34]. HIV-related stigma was measured using the HIV Stigma Scale, which includes 7 items on a 4-point Likert scale, such as “I feel guilty because I have HIV.” The total score ranges from 14 to 56, with high scores indicating high levels of HIV-related stigma [35]. The 9-item Patient Health Questionnaire (PHQ-9) is a widely used criteria-based diagnostic tool for depressive symptoms. It was used to assess depression severity over the past 2 weeks using a 3-point Likert scale ranging from *not at all* to *nearly every day*. The total score ranges from 0 to 27, with score ≥5 being considered as having depressive symptoms [36]. We used metabolic equivalents, which were calculated using the Chinese version of the Global Physical Activity Questionnaire to describe the intensity of physical activities of the participants [37].

### Statistical Analysis

The intention-to-treat principle was applied to all the analyses. Descriptive statistics for baseline characteristics and psychological outcomes (eg, CES-D, QOL, and PSS scores) were provided. Continuous variables were described as means and SDs for normally distributed variables or medians and IQRs for nonnormally distributed variables and frequencies and percentages for categorical variables. Between-group differences at baseline were reported using the independent samples 2-tailed *t* test, chi-square (*χ^2^*) test, or nonparametric test, as appropriate, and the 95% CIs were calculated. Similar analyses were performed for group differences between participants who completed the 1-year or 3-year follow-up and those who dropped out at the 1-year or 3-year follow-up. For missing values, multivariate imputation by chained equations was performed using R package mice (version 4.0.5; R Foundation for Statistical Computing), and 80 imputed data sets were obtained. The final data set was the average of the 80 imputed data sets. All the statistical analyses were conducted using the complete data set after imputation. In addition, sensitivity analysis was conducted using data with missing values to assess the robustness of the results.

For primary and secondary outcome analyses, the repeated measures generalized estimating equation (GEE) linear regression models were used to assess the intervention effects [38]. As a model that is well suited for longitudinal data analysis, GEE improves statistical power because it allows for the simultaneous analysis of intervention effects at multiple time points in a single model, with an exchangeable working correlation matrix accounting for potential correlation owing to within-participant dependencies across time. In this study, the main effects of group and time and the interaction effects between group and time were examined using the GEE, using repeated measurements of the 2 groups at baseline and the 5 follow-up points (ie, 3-, 6-, 9-, 12-, and 36-month follow-ups), adjusting for baseline demographic and HIV-related characteristics (ie, age, sex, BMI, education, sexual orientation, family monthly income, marital status, duration of HIV infection, and employment) and psychological outcomes (eg, CES-D scores). The R package geepack (version 4.0.5) was used to conduct the GEE analysis.

Cohen *d* was calculated to measure the effect size of the intervention [39]. The between-group effect size was calculated using the difference between the mean score change of the intervention group from baseline and that of the control group from baseline, which was then divided by the SD of the pooled score changes. Cohen *d* >0.20 was considered as a small effect size, Cohen *d* >0.50 as a medium effect size, and Cohen *d* >0.80 as a large effect size [40].

All analyses were performed using R (version 4.0.5; R Foundation for Statistical Computing). All statistical tests were 2-sided, and *P*<.05 was considered as statistically significant.

## Results

### Sample Characteristics

**Figure 1:**
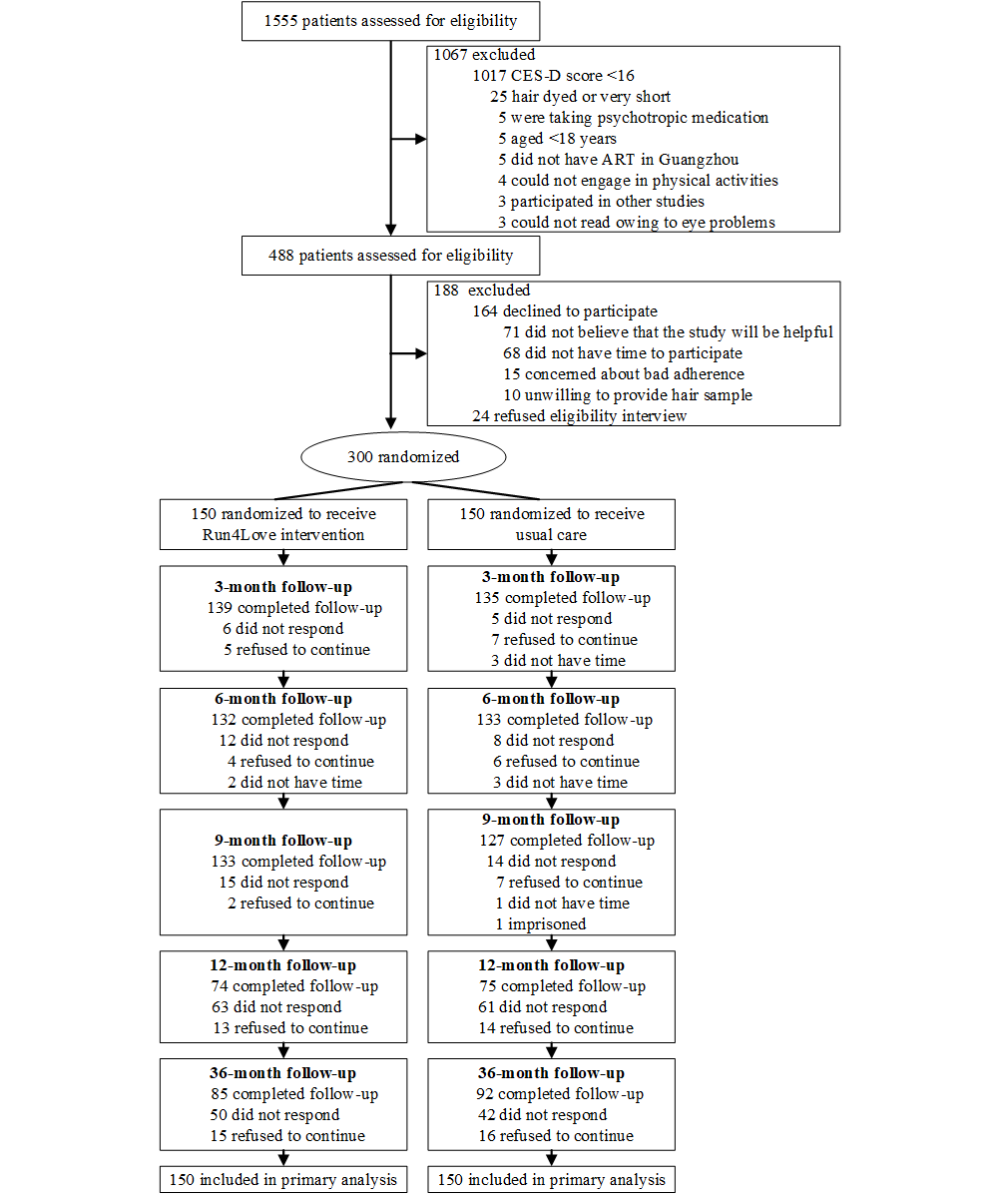
Flow chart of the Run4Love trial and the long-term follow-ups. ART: antiretroviral therapy; CES-D: Center for Epidemiological Studies–Depression.

Among the 300 participants, approximately half (n=149, 49.7%) completed the 1-year follow-up evaluation, including 49.7% (74/149) of them in the Run4Love intervention group and 50.3% (75/149) of them in the control group. A total of 59% (177/300) of the participants completed the 3-year follow-up evaluation, including 48% (85/177) of them in the intervention group and 51.9% (92/177) of them in the control group (Figure 1). The baseline characteristics of the participants who completed the baseline, 1-year, and 3-year follow-up surveys are shown in Table 1. All demographic characteristics were balanced between the 2 groups at baseline, except sexual orientation, sex, and family monthly income. The mean completion rate of the CBSM courses was 55% in the Run4Love intervention group and 4% in the wait-list control group.

Similarly, the baseline characteristics were compared between participants who completed the assessments and those who were lost to follow-up. There were significant differences in BMI, duration of HIV infection, and self-efficacy at baseline between participants who completed the 1-year follow-up questionnaire and those who were lost to follow-up. Significant differences were also found in age, BMI, and duration of HIV infection at baseline between participants who completed the 3-year follow-up questionnaire and those who were lost to follow-up (Tables S1 and S2 in Multimedia Appendix 2). Baseline characteristics of participants who did not complete the 1-year or 3-year outcome evaluation between the 2 groups were comparable (Tables S3 and S4 in Multimedia Appendix 2).

**Table 1:** Baseline characteristics of participants in the intervention and control groups.

Baseline characteristics	Baseline		1-year follow-up		3-year follow-up	
	Run4Love group (n=150)	Usual care group (n=150)	Run4Love group (n=74)	Usual care group (n=75)	Run4Love group (n=85)	Usual care group (n=92)
Age (years), mean (SD)	28 (5.8)	28.6 (5.9)	28.1 (5.5)	27.9 (6.1)	28.1 (5.3)	27.4 (5.5)
Sex (male), n (%)	142 (94.7)	135 (90)	73 (99)	67 (89)	82 (96)	84 (91)
BMI^a^, mean (SD)	20.5 (2.5)	20.1 (2.4)	19.5 (1.7)	19.3 (1.5)	19.5 (1.9)	19.7 (2.3)
Education level greater than high school, n (%)	98 (65.3)	84 (56)	51 (69)	44 (59)	59 (69)	52 (57)
Homosexual, bisexual, or uncertain, n (%)	130 (86.7)	115 (76.7)	67 (91)	60 (80)	75 (88)	73 (79)
Married, n (%)	18 (12)	20 (13.3)	6 (8)	7 (9)	8 (9)	10 (11)
Family monthly income 7000 Yuan (US $1043.7), n (%)	68 (45.3)	56 (37.3)	32 (43)	24 (32)	44 (52)	27 (29)
Duration of HIV infection, median (IQR)	1.7 (0.6-4)	1.7 (0.6-3.6)	1.2 (0.5-1.7)	1.2 (0.9-2.3)	1.2 (0.7-1.7)	1.2 (0.4-2)
CES-D^b^ score, mean (SD)	23.9 (6.4)	24.3 (6.9)	23.8 (5.9)	24.7 (7.7)	24.5 (6.2)	24.5 (7)
PHQ-9^c^ score, mean (SD)	10.2 (4.5)	10.7 (5.1)	10 (4.1)	10.8 (5.7)	10.5 (4.7)	11 (5.6)
WHOQOL-HIV BREF^d^ score, mean (SD)	77.4 (9)	76.6 (9.4)	77.5 (8.3)	75.3 (10.4)	76.9 (8.6)	76.3 (10.2)
GSES^e^ score, mean (SD)	24.4 (5.2)	23.3 (5.6)	23.6 (5.2)	22.3 (5.8)	24 (4.9)	23 (5.9)
PSS^f^ score, mean (SD)	20 (4.4)	20.7 (4.4)	19.8 (4.4)	21.4 (4.8)	20.1 (4.6)	21.1 (4.8)
HIV Stigma Scale score, mean (SD)	37.1 (7.7)	38 (7.5)	36.5 (7.7)	37.5 (9)	36.7 (8)	38.1 (8.5)
SWCQ positive coping^g^ score, mean (SD)	18.4 (5.5)	18.3 (6.2)	18.1 (4.7)	18.1 (6.3)	18.6 (5.2)	18.7 (6.1)
SWCQ negative coping^h^ score, mean (SD)	11.8 (3.9)	11.8 (3.9)	11.3 (3.9)	12.1 (4.1)	11.5 (4)	11.9 (4)
Physical activity (metabolic equivalents^i^ ≥600), n (%)	65 (43.3)	65 (43.3)	43 (58)	44 (59)	46 (54)	49 (53)

^a^Calculated as weight in kilograms divided by height in meters squared.

^b^CES-D: Center for Epidemiological Studies–Depression.

^c^PHQ-9: 9-item Patient Health Questionnaire.

^d^WHOQOL-HIV BREF: World Health Organization Quality of Life HIV short version.

^e^GSES: General Self-Efficacy Scale.

^f^PSS: Perceived Stress Scale.

^g^SWCQ positive coping: Simplified Ways of Coping Questionnaire positive coping domain.

^h^SWCQ negative coping: SWCQ negative coping domain.

^i^Physical activity was measured by metabolic equivalents. Metabolic equivalents ≥600 indicates recommended physical activity level.

### Primary Outcome at 1-Year Follow-up

As previously reported, significant reductions in the primary outcome (depressive symptoms) were observed among participants in the intervention group at the 3-, 6-, and 9-month follow-ups [16]. The sustained effects of reduced depressive symptoms were further observed at 1-year follow-up. At the 1-year follow-up, the Run4Love intervention group showed significant reduction in the CES-D scores compared with that shown by the control group (from 23.9 to 18.1 in the intervention group vs from 24.3 to 23.3 in the control group; mean difference between groups −4.79, SD 13.56; 95% CI −7.78 to −1.81; *P*=.002), with standard effect size (Cohen *d*) of 0.48 in favor of the Run4Love intervention (Table 2).

**Table 2:** Long-term effects of the Run4Love intervention on primary and secondary outcomes.

Outcomes and time points			Run4Love intervention group (n=150)					Usual care group (n=150)					Between-group difference for mean change from baseline, mean difference (95% CI)			*P* value^a^
			Follow-up, mean (SD)		Within-group changes, mean difference (95% CI)^b^		Follow-up, mean (SD)			Within-group changes, mean difference (95% CI)						
**Depressive symptoms (Center for Epidemiological Studies–Depression scale^c^)**																
	Baseline	23.9 (6.4)		N/A^d^		24.3 (6.9)			N/A		N/A			N/A		
	1-year follow-up	18.1 (11.3)		−5.79 (−7.99 to −3.59)		23.3 (12.4)			−0.99 (−3.27 to 1.29)		−4.79 (−7.78 to −1.81)			.002		
	3-year follow-up	20.5 (11.2)		−3.47 (−5.64 to −1.31)		24.4 (12.7)			0.15 (−2.07 to 2.38)		−3.63 (−6.71 to −0.54)			.02		
**Quality of life^e^**																
	Baseline	77.4 (9)		N/A		76.6 (9.4)			N/A		N/A			N/A		
	1-year follow-up	82.8 (14.1)		5.42 (2.88 to 7.95)		77.9 (14.7)			1.26 (−1.08 to 3.6)		4.15 (0.81 to 7.50)			.02		
	3-year follow-up	78.6 (13.7)		1.13 (−1.23 to 3.48)		74.1 (14.3)			−2.50 (−4.96 to −0.05)		3.63 (0.34 to 6.92)			.03		
**Perceived stress (Perceived Stress Scale^c^)**																
	Baseline	20 (4.4)		N/A		20.7 (4.4)			N/A		N/A			N/A		
	1-year follow-up	16.6 (6.1)		−3.38 (−4.70 to −2.06)		19.3 (6.6)			−1.41 (−2.74 to −0.08)		−1.97 (−3.67 to −0.26)			.02		
	3-year follow-up	18 (5.9)		−1.93 (−3.04 to −0.81)		19.9 (6.6)			−0.80 (−1.99 to 0.38)		−1.12 (−2.73 to 0.48)			.17		
**Simplified Ways of Coping Questionnaire positive coping^e^**																
	Baseline	18.4 (5.5)		N/A		18.3 (6.2)			N/A		N/A			N/A		
	1-year follow-up	21 (7.6)		2.59 (0.95 to 4.23)		18.2 (7.1)			−0.16 (−1.59 to 1.27)		2.75 (0.76 to 4.74)			.007		
	3-year follow-up	19.2 (6.9)		0.78 (−0.84 to 2.4)		17.8 (6.4)			−0.53 (−1.84 to 0.79)		1.31 (−0.72 to 3.34)			.21		
**Simplified Ways of Coping Questionnaire negative coping^c^**																
	Baseline	11.8 (3.8)		N/A		11.7 (3.9)			N/A		N/A			N/A		
	1-year follow-up	11.9 (4.3)		0.11 (−0.81 to 1.03)		11.9 (4.6)			0.19 (−0.76 to 1.15)		−0.08 (−1.38 to 1.21)			.90		
	3-year follow-up	12.1 (4)		0.29 (−0.67 to 1.25)		11.6 (4)			−0.11 (−1.01 to 0.79)		0.40 (−0.91 to 1.70)			.55		
**Self-efficacy (General Self-Efficacy Scale^e^)**																
	Baseline	24.4 (5.2)		N/A		23.3 (5.6)			N/A		N/A			N/A		
	1-year follow-up	26.6 (6.7)		2.22 (0.82 to 3.62)		23.5 (6.4)			0.20 (−1.12 to 1.51)		2.02 (0.19 to 3.85)			.03		
	3-year follow-up	25.1 (6.4)		0.73 (−0.52 to 1.98)		22.9 (6.5)			−0.42 (−1.72 to 0.87)		1.15 (−0.61 to 2.92)			.19		
**HIV Stigma Scale^c^**																
	Baseline	37.1 (7.7)		N/A		38 (7.5)			N/A		N/A			N/A		
	1-year follow-up	33.5 (9.6)		−3.62 (−5.43 to −1.82)		36.9 (10.4)			−1.10 (−2.95 to 0.76)		−2.53 (−5.17 to 0.09)			.06		
	3-year follow-up	35.4 (9.9)		−1.70 (−3.43 to 0.03)		37.6 (10.1)			−0.43 (−2.14 to 1.28)		−1.27 (−3.67 to 1.12)			.30		
**Depression severity (9-item Patient Health Questionnaire^c^)**																
	Baseline	10.2 (4.5)		N/A		10.7 (5.1)			N/A		N/A			N/A		
	1-year follow-up	7.2 (5.1)		−2.98 (−4.09 to −1.87)		8.5 (5.5)			−2.23 (−3.30 to −1.15)		−0.75 (−2.21 to 0.71)			.31		
	3-year follow-up	8.4 (5.4)		−1.75 (−2.87 to −0.63)		8.7 (5.9)			−2.06 (−3.16 to −0.97)		0.31 (−1.24 to 1.87)			.69		
**Physical activity (metabolic equivalents^e^)**																
	Baseline	3225 (6189)		N/A		2675 (6064)			N/A		N/A			N/A		
	1-year follow-up	4193 (20,017)		969 (−3414 to 5352)		6850 (37,316)			4176 (−2041 to 10,393)		−3206 (−10,734 to 4320)			.40		
	3-year follow-up	2788 (5470)		−436 (−1814 to 941)		2933 (6860)			258 (−1142 to 1660)		−695 (−2674 to 1283)			.49		

^a^Results based on the generalized estimating equations, adjusted for age, sex, BMI, education, sexual orientation, family monthly income, marital status, duration of HIV infection, and employment.

^b^Within-group changes are mean changes.

^c^High score indicates worse outcome.

^d^N/A: not applicable.

^e^High score indicates better outcome.

### Primary Outcome at 3-Year Follow-up

At the 3-year follow-up, between-group difference in the CES-D scores remained statistically significant (mean difference between groups −3.63, SD 13.35; 95% CI −6.71 to −0.54; *P*=.02; Cohen *d*=0.36). Results from the GEE model on the long-term intervention effects on depressive symptoms at the 1-year and 3-year follow-ups are summarized in Table 2. Results indicated that there was no main effect of group or time, but there were significant interaction effects between group and time, with statistically significant between-group differences in the CES-D score for mean changes from baseline, after controlling for baseline characteristics. The results were similar to those obtained from data without multiple imputations for missing values (Table S5 in Multimedia Appendix 2). Significant between-group differences were observed at both 1-year and 3-year follow-up. Results from all the measurement time points (ie, 3, 6, and 9 months and 1 and 3 years), GEE model, and sensitivity analyses showed similar findings (Tables S6-S8 in Multimedia Appendix 2).

**Figure 2:**
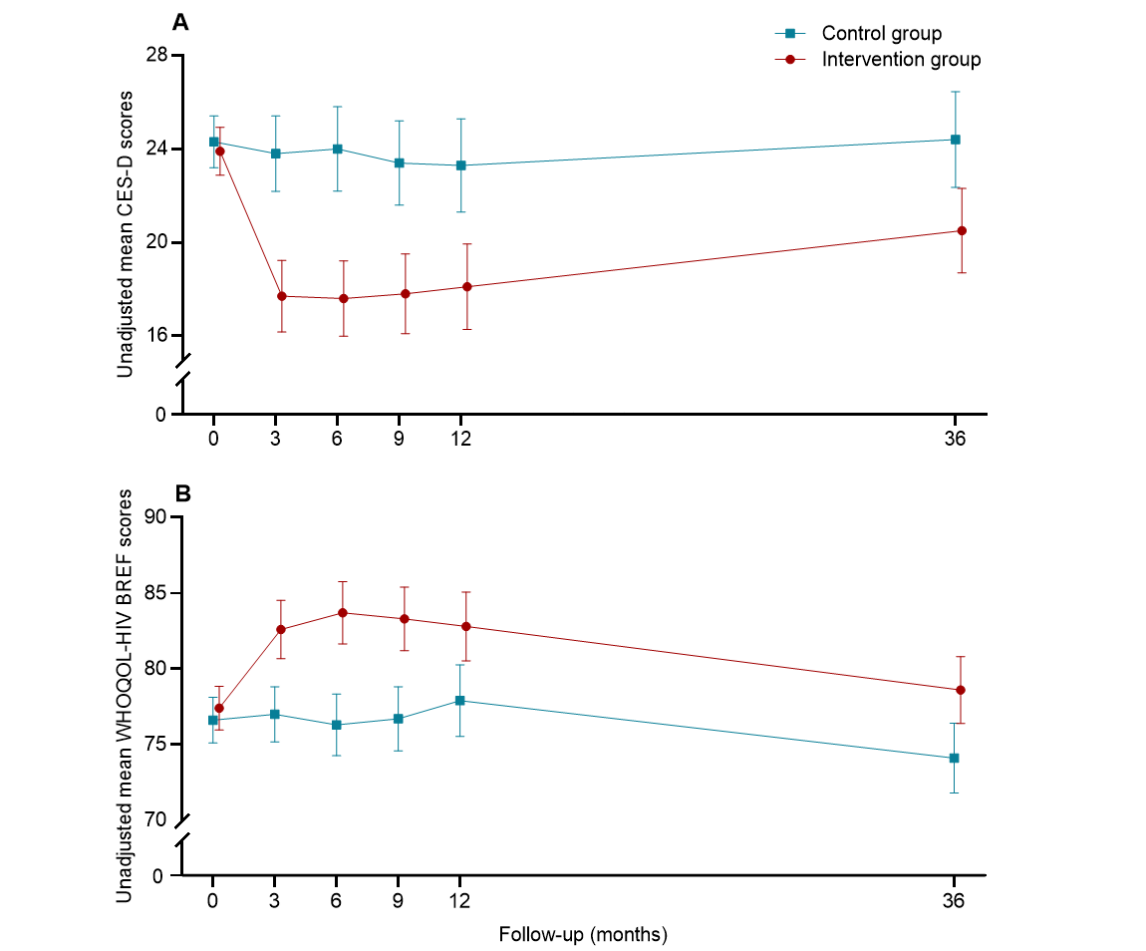
Measures of depressive symptoms (Center for Epidemiological Studies–Depression [CES-D] scale) and quality of life (World Health Organization Quality of Life HIV short version [WHOQOL-HIV BREF]) at baseline and follow-ups in the Run4Love intervention and control groups.

Change patterns of depressive symptoms (CES-D) at baseline and follow-ups in the 2 groups are presented in Figure 2. During the 3-year follow-up period, the average CES-D scores of the control group remained fairly stable. By contrast, the average CES-D scores of the intervention group decreased significantly after the Run4Love intervention at 3 months. After the largest decline in depressive symptoms at 3 months, the CES-D scores in the intervention group gradually increased, but never reached as high as they were at baseline, and the between-group differences remained statistically significant throughout the 3-year follow-up.

### Secondary Outcomes at 1-Year Follow-up

**Figure 3:**
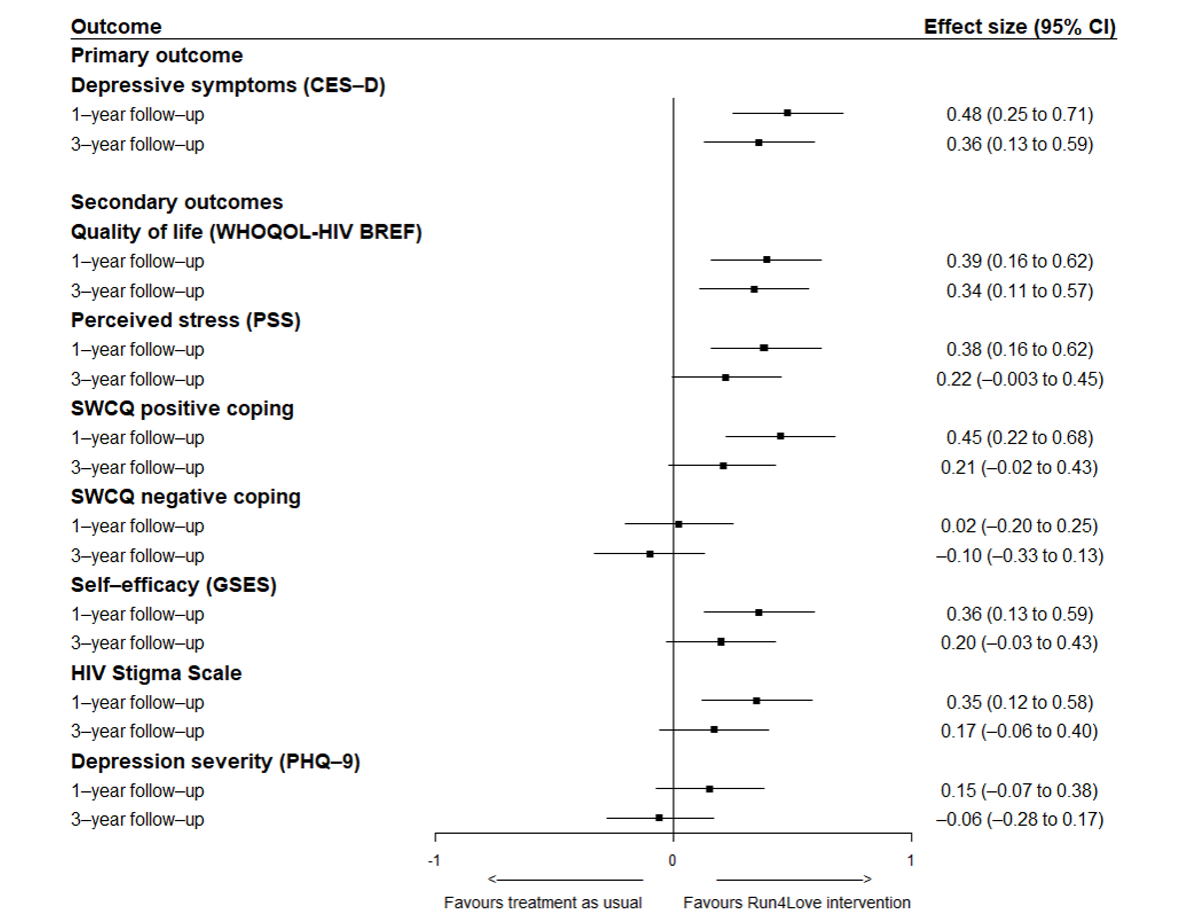
Plot showing the Cohen d effect sizes and 95% CIs for the primary and secondary outcomes of the Run4Love intervention group versus the control group. CES-D: Center for Epidemiological Studies–Depression; GSES: General Self-Efficacy Scale; PHQ-9: 9-item Patient Health Questionnaire; PSS: Perceived Stress Scale; SWCQ: Simplified Ways of Coping Questionnaire; WHOQOL-HIV BREF: World Health Organization Quality of Life HIV short version

Results of the long-term intervention effects on secondary outcomes are presented in Table 2. At the 1-year follow-up, compared with the control group, participants in the Run4Love intervention group reported significantly improved QOL (WHOQOL-HIV BREF: from 77.4 to 82.8 in the intervention group vs 76.6 to 77.9 in the control group; mean difference between groups 4.15, SD 15.61; 95% CI 0.81-7.50; *P*=.02), with standard effect size of 0.39 in favor of the Run4Love intervention group (Table 2; Figure 3). Similarly, participants in the intervention group also reported significantly reduced perceived stress (PSS: from 20 to 16.6 in the intervention group vs from 20.7 to 19.3 in the control group; mean difference between groups –1.97, SD 8.10; 95% CI −3.67 to −0.26; *P*=.02) and improved SWCQ positive coping (from 18.4 to 21 in the intervention group vs from 18.3 to 17.8 in the control group; mean difference between groups 2.75, SD 10.10; 95% CI 0.76-4.74; *P*=.007) and self-efficacy (General Self-Efficacy Scale: from 24.4 to 26.6 in the intervention group vs from 23.3 to 23.4 in the control group; mean difference between groups 2.02, SD 8.63; 95% CI 0.19-3.85; *P*=.03). There were no significant between-group differences in changes in SWCQ negative coping, HIV-related stigma (HIV Stigma Scale), depression severity (PHQ-9), and physical activity (metabolic equivalents) at 1 year. The results were also similar to those obtained from data without multiple imputations for missing values (Table S5 in Multimedia Appendix 2).

### Secondary Outcomes at 3-Year Follow-up

At the 3-year follow-up, the intervention effect remained statistically significant for QOL (WHOQOL-HIV BREF: from 77.4 to 78.6 in the intervention group vs 76.6 to 74.1 in the control group; mean difference between groups 3.63, SD 14.53; 95% CI 0.34-6.92; *P*=.03; Cohen *d*=0.34). There were no significant between-group differences in the changes in other measures of the secondary outcomes. The results were similar to those obtained from data without multiple imputations for missing values (Table S5 in Multimedia Appendix 2).

Regarding the change patterns of QOL, in the first 12 months, the average QOL scores in the control group remained moderately stable and declined slightly at the 3-year follow-up. By contrast, significant increase in the average QOL scores was observed in the intervention group at 3 months, immediately upon completion of the Run4Love program, and the score continued to increase, with the largest group difference occurring at the 6-month follow-up. Although the QOL scores in the intervention group gradually and slowly decreased over time, significant between-group differences were observed at 9-month, 1-year, and 3-year follow-ups (Figure 2). Change patterns of other secondary outcomes are summarized in Figure S1 in Multimedia Appendix 2.

## Discussion

### Principal Findings

This study is among the first efforts to investigate the long-term effects of an mHealth intervention on depressive symptoms among people living with HIV, with a follow-up period of 3 years. Results showed that, compared with the control, the Run4Love intervention significantly reduced depressive symptoms (CES-D) and improved QOL not only at 3, 6, and 9 months but also at 1-year and 3-year follow-ups [16]. In addition, changes in secondary outcomes including perceived stress, positive coping, and self-efficacy remained significant at the 1-year follow-up, but the between-group differences in these measures were not significant at the 3-year follow-up.

### Results Interpretation and Implication

This study addresses several gaps in the literature regarding long-term effects of mHealth interventions on depressive symptoms in people living with HIV. Few studies have investigated the long-term effects (>1 year) of mHealth interventions on depressive symptoms among people living with HIV [12-15,41-45]. A study in the Netherlands found that a 2-month internet-based intervention using cognitive behavioral therapy effectively reduced depressive symptoms among people living with HIV [24]. However, the Netherlands-based study followed the intervention group for 8 months from baseline and the control group for only 5 months. The study found sustained intervention effect on depressive symptoms over time. The only study that we found on mHealth interventions targeting depressive symptoms among people living with HIV, with a follow-up period of ≥1 year was conducted by Li et al [45]. They explored the impact of 1-month positive psychology and social networking intervention on depressive symptoms of HIV-infected men who have sex with men in Chengdu, China, but the results showed no significant improvement in depressive symptoms at the 13-month follow-up.

Compared with mHealth interventions, in-person interventions have shown more sustained long-term effects on depressive symptoms among people living with HIV [46,47]. A total of 2 in-person intervention studies reported significant intervention effects at 1-year follow-up. Steven et al [46] showed that an 11-week cognitive behavioral therapy effectively reduced depressive symptoms among people living with HIV, and the effects were sustained for up to 12 months. Another study of 8 weekly sessions, which lasted 2 to 3 hours each, based on group support psychotherapy also achieved significant improvement in depressive symptoms among people living with HIV [47]. Although effective, these in-person psychotherapy programs are very expensive and difficult to scale up, especially in resource-poor settings. Furthermore, few of these in-person interventions have reported long-term effects for up to 3 years.

From previous qualitative interviews, secondary data analyses, and researchers’ experience, we offer some possible explanations for the significant long-term intervention effects found in this study: theory-guided intervention design, combination of web-based and offline interactions, continuous process monitoring, and changed perception and behaviors among the participants. First, the Run4Love program was adapted from evidence-based CBSM courses that are effective in reducing depressive symptoms among people living with HIV [46,48]. Second, the Run4Love intervention combined both offline interactions, between researchers and participants at baseline and 3, 6, and 9 months, and web-based interactions, such as reminders for course completion and 5 phone calls at 1 week and 1, 2, 5, and 8 months from baseline. A trusting relationship between participants and researchers was built at the beginning of the program and maintained over time, which was critical for the success of the intervention [49]. Third, continuous process monitoring ensured a satisfactory level of patient engagement, which is essential for the effectiveness of an mHealth intervention [50]. On average, participants in the intervention group completed 55% of the CBSM coursework, which was comparable with that in other mHealth interventions, whereas those in the wait-list control group completed only 4% of the coursework, as they received no regular monitoring and phone calls [51,52]. Finally, through feedback at the 3-year follow-up, participants shared their personal experience of how their perception of stress changed over time and how they adapted their coping behaviors as a result of the cognitive changes during and after the Run4Love intervention. They also commented that specific stress-reduction skills such as exercise, relaxation, and meditation were very helpful in managing depressive symptoms.

Notably, participants in the intervention group showed significant reduction in depressive symptoms measured by CES-D scores, which was sustained at the 3-year follow-up; however, the between-group differences in PHQ-9 scores were only significant at the 3-month and 6-month follow-ups and were not significant at later follow-ups. This discrepancy may be because of the differences between these 2 measurements. The PHQ-9 is more widely used for depression screening, whereas the CES-D measure has high sensitivity, specificity, and responsiveness in detecting and monitoring changes in depressive symptoms in longitudinal studies compared with the PHQ-9 [53,54]. In addition, the positive effects of physical activity on mental health improvement, including reducing depressive symptoms, are well established in the literature [55,56]; however, we did not observe significant improvement in physical activity in our RCT. It may be possible that the intervention was not effective in improving participants’ physical activity or that we did not capture changes in physical activity appropriately and effectively, as physical activity was self-reported. Although in our qualitative interviews at the 3-year follow-up, some participants mentioned that they exercised regularly because of our intervention, the effects of physical activity promotion in our intervention are unclear.

It is imperative and beneficial to promote mHealth interventions with long-term effects on depressive symptoms among people living with HIV, especially during the COVID-19 pandemic, which not only exacerbates distress in vulnerable populations but also limits their access to health services. mHealth interventions offer a promising alternative to in-person care in the delivery of urgently needed mental health services in underserved communities or areas with shortage of mental health professionals, such as Africa or other low-income and middle-income countries or regions. Furthermore, as depressive symptoms are a common comorbidity with many chronic diseases including HIV infection, evidence-based mHealth interventions such as Run4Love may be adapted and integrated into routine care or depressive symptom management.

### Limitations

This study has several limitations. First, the dropout rates at the 1-year and 3-year follow-ups were relatively high, which is a common challenge in long-term follow-ups in interventions, especially in mHealth interventions [57,58]. However, we managed to trace more participants at 3-year follow-up than 1-year follow-up. To examine the robustness of the results at the 1-year and 3-year follow-ups, we used sensitivity analyses and found similar and robust results using data with missing values and data without missing values after multiple imputations. Second, all the participants were recruited from a large hospital in a capital city in South China, and most of them were men, especially young men; therefore, the results from this study may not be generalizable to people living with HIV living in rural areas or women who live with HIV.

### Conclusions

In conclusion, this study found that a social media–based mHealth intervention, Run4Love, significantly reduced depressive symptoms and improved QOL among people living with HIV at 1-year and 3-year follow-ups. Further research is needed to explore the mechanisms underlying the long-term effects of mHealth interventions and understand how to implement evidence-based interventions with sustained effects to better serve people living with HIV with depression.

## Appendix

Multimedia Appendix 1 CONSORT-eHEALTH checklist (V 1.6.1).

Multimedia Appendix 2 Sensitivity analyses of results on primary and secondary outcomes.

## Abbreviations

CBSM: cognitive behavioral stress management
CES-D: Center for Epidemiological Studies–Depression
CONSORT–EHEALTH: Consolidated Standards of Reporting Trials of Electronic and Mobile Health Applications and Online Telehealth
GEE: generalized estimating equation
mHealth: mobile health
PHQ-9: 9-item Patient Health Questionnaire
PSS: Perceived Stress Scale
QOL: quality of life
RCT: randomized controlled trial
SWCQ: Simplified Ways of Coping Questionnaire
WHOQOL-HIV BREF: World Health Organization Quality of Life HIV short version
