# Supplementary material for: Long-term Effects of a Social Media–Based Intervention (Run4Love) on Depressive Symptoms of People Living With HIV: 3-Year Follow-up of a Randomized Controlled Trial
Source: J Med Internet Res. 2022 Jun 28;24(6):e36809. doi: 10.2196/36809 (PMC9277532; doi:10.2196/36809)
Supplement: Multimedia Appendix 2 [file jmir_v24i6e36809_app2.docx]

**Supplementary Tables and Figures:**

Table S1. Comparison of Baseline Characteristics of Participants between Those Who Were Lost at the 1-year Assessment and Who Completed the 1-year Outcome Assessment

Table S2. Comparison of Baseline Characteristics of Participants between Those Who Were Lost at the 3-year Assessment and Who Completed the 3-year Outcome Assessment

Table S3. Comparison of Baseline Characteristics between the Run4Love group and the Usual Care Group among Participants Who did not Complete the 1-year Outcome Evaluation

Table S4. Comparison of Baseline Characteristics between the Run4Love group and the Usual Care Group among Participants Who did not Complete the 3-year Outcome Evaluation

Table S5. Effects of the Run4Love Intervention on the Primary and Secondary Outcomes Without Missing Data Imputation

Table S6. Effects of the Run4Love Intervention on the Primary and Secondary Outcomes (Full Results)

Table S7. GEE Results of the Primary Outcome and Secondary Outcomes (Full Results)

Table S8. GEE Results of the Primary Outcome and Secondary Outcomes Without Missing Data Imputation

Figure S1. Secondary Outcomes Over Time for the Run4Love Intervention Group vs Control Group

**Table S1. Comparison of Baseline Characteristics of Participants between Those Who Were Lost at the 1-year Assessment and Who Completed the 1-year Outcome Assessment**

| **Variables** | **Lost before the 1-year evaluation (n=151)** | **Completed the 1-year outcome evaluation (n=149)** | ***P* value** |
| --- | --- | --- | --- |
| Age, mean (SD) | 28.7 (5.9) | 28.0 (5.8) | .31 |
| Gender (men), n (%) | 137 (90.7) | 140 (94.0) | .29 |
| BMI, mean (SD) | 20.4 (2.7) | 19.4 (1.6) | <.001 |
| Education level greater than high school, n (%) | 87 (57.6) | 95 (63.8) | .28 |
| Homosexual or bisexual or uncertain, n (%) | 118 (78.1) | 127 (85.2) | .11 |
| Married, n (%) | 24 (15.9) | 13 (8.7) | .06 |
| Family monthly income ≥ 7000 Yuan (US $1043.7), n (%) | 67(44.4) | 56 (37.6) | .23 |
| Duration of HIV infection, median (IQR) | 1.49 (0.53 to 3.17) | 1.17 (0.62 to 2.34) | .05 |
| CES-D score, mean (SD) | 23.9 (6.4) | 24.2 (6.9) | .70 |
| WHOQOL-HIV BREF score, mean (SD) | 77.6 (9.0) | 76.4 (9.5) | .26 |
| PSS score, mean (SD) | 20.1 (4.2) | 20.6 (4.6) | .31 |
| SWCQ positive coping score, mean (SD) | 18.6 (6.0) | 18.1 (5.6) | .52 |
| SWCQ negative coping score, mean (SD) | 11.8 (3.7) | 11.7 (4.0) | .73 |
| GSES score, mean (SD) | 24.8 (5.2) | 22.9 (5.5) | .002 |
| HIV Stigma Scale score, mean (SD) | 38.1 (6.8) | 37.0 (8.4) | .23 |
| PHQ-9 score, mean (SD) | 10.4 (4.7) | 10.4 (5.0) | .97 |
| METs, median (IQR) | 840 (0 to 2400) | 960 (140 to 2460) | .65 |
| Abbreviations: SD, standard deviation; IQR, interquartile range; BMI, Body Mass Index; CES-D, the Center for Epidemiological Studies-Depression Scale; QOL, quality of life; GSES, General Self-efficacy Scale; PSS, Perceived Stress Scale; SWCQ, Simplified Ways of Coping Questionnaire; PHQ-9, 9-item Patient Health Questionnaire; METs, Metabolic equivalents. | | | |

**Table S2. Comparison of Baseline characteristics of Participants between Those Who Were Lost at the 3-year Assessment and Who Completed the 3-year Outcome Assessment**

| **Variables** | **Lost before the 3-year evaluation (n=123)** | **Completed the 3-year outcome evaluation (n=177)** | ***P* value** |
| --- | --- | --- | --- |
| Age, mean (SD) | 29.1 (6.4) | 27.7 (5.4) | .04 |
| Gender (men), n (%) | 111 (90.2) | 166 (93.8) | .26 |
| BMI, mean (SD) | 20.3 (2.5) | 19.6 (2.1) | .007 |
| Education level greater than high school, n (%) | 71 (57.7) | 111 (62.7) | .38 |
| Homosexual or bisexual or uncertain, n (%) | 97 (78.9) | 148 (83.6) | .30 |
| Married, n (%) | 19 (15.4) | 18 (10.2) | .17 |
| Family monthly income ≥ 7000 Yuan (US $1043.7), n (%) | 52 (42.3) | 71 (40.1) | .71 |
| Duration of HIV infection, median (IQR) | 1.71 (0.53 to 4.25) | 1.17 (0.72 to 1.84) | .003 |
| CES-D score, mean (SD) | 23.5 (6.7) | 24.5 (6.6) | .21 |
| WHOQOL-HIV BREF score, mean (SD) | 77.7 (8.8) | 76.6 (9.5) | .31 |
| PSS score, mean (SD) | 19.9 (4.0) | 20.6 (4.7) | .18 |
| SWCQ positive coping score, mean (SD) | 17.9 (6.0) | 18.7 (5.6) | .28 |
| SWCQ negative coping score, mean (SD) | 11.8 (3.6) | 11.7 (4.0) | .83 |
| GSES score, mean (SD) | 24.4 (5.4) | 23.5 (5.4) | .14 |
| HIV Stigma Scale score, mean (SD) | 37.7 (6.5) | 37.4 (8.3) | .76 |
| PHQ-9 score, mean (SD) | 10.0 (4.2) | 10.8 (5.2) | .16 |
| METs, median (IQR) | 720 (0 to 2400) | 1000 (240 to 2580) | .21 |
| Abbreviations: SD, standard deviation; IQR, interquartile range; BMI, Body Mass Index; CES-D, the Center for Epidemiological Studies-Depression Scale; QOL, quality of life; GSES, General Self-efficacy Scale; PSS, Perceived Stress Scale; SWCQ, Simplified Ways of Coping Questionnaire; PHQ-9, 9-item Patient Health Questionnaire; METs, Metabolic equivalents. | | | |

**Table S3 Comparison of Baseline Characteristics between the Run4Love group and the Usual Care Group among Participants Who did not Complete the 1-year Outcome Evaluation**

| **Variables** | **Run4love group**  **(n=76)** | **Usual care group**  **(n=75)** | ***P* value** | |
| --- | --- | --- | --- | --- |
| Age, mean (SD) | 28.0 (6.11) | 29.3 (5.56) | .16 | |
| Gender (men), n (%) | 69 (91) | 68 (91) | >.99 | |
| BMI, mean (SD) | 20.4 (2.79) | 20.4 (2.65) | .85 | |
| Education level greater than high school, n (%) | 47 (62) | 40 (53) | .37 | |
| Homosexual or bisexual or uncertain, n (%) | 63 (83) | 55 (73) | .22 | |
| Married, n (%) | 11 (15) | 13 (17) | .80 | |
| Family monthly income ≥ 7000 Yuan (US $1043.7), n (%) | 38 (50) | 29 (39) | .22 | |
| Duration of HIV infection, median (IQR) | 1.38 (0.65 to 3.74) | 1.64 (0.41 to 3.00) | .76 | |
| CES-D score, mean (SD) | 24.1 (6.9) | 23.8 (5.9) | .81 | |
| WHOQOL-HIV BREF score, mean (SD) | 77.3 (9.7) | 77.9 (8.2) | .72 | |
| PSS score, mean (SD) | 20.1 (4.5) | 20.0 (3.9) | .89 | |
| SWCQ positive coping score, mean (SD) | 18.6 (6.1) | 18.5 (6.0) | .90 | |
| SWCQ negative coping score, mean (SD) | 12.3 (3.8) | 11.4 (3.6) | .15 | |
| GSES score, mean (SD) | 25.2 (5.1) | 24.4 (5.3) | .32 | |
| HIV Stigma Scale score, mean (SD) | 37.7 (7.6) | 38.5 (5.7) | .46 | |
| PHQ-9 score, mean (SD) | 10.3 (5.0) | 10.6 (4.4) | .63 | |
| METs, median (IQR) | 654 (0 to 2430) | 1200 (260 to 2400) | .42 | |
| Abbreviations: SD, standard deviation; IQR, interquartile range; BMI, Body Mass Index; CES-D, the Center for Epidemiological Studies-Depression Scale; QOL, quality of life; GSES, General Self-efficacy Scale; PSS, Perceived Stress Scale; SWCQ, Simplified Ways of Coping Questionnaire; PHQ-9, 9-item Patient Health Questionnaire; METs, Metabolic equivalents. | | | |  |

**Table S4 Comparison of Baseline Characteristics between the Run4Love group and the Usual Care Group among Participants Who did not Complete the 3-year Outcome Evaluation**

| **Variables** | **Run4love group**  **(n=65)** | **Usual care group (n=58)** | ***P* value** |
| --- | --- | --- | --- |
| Age, mean (SD) | 28.0 (6.5) | 30.5 (6.0) | .03 |
| Gender (men), n (%) | 60 (92) | 51 (88) | .61 |
| BMI, mean (SD) | 20.4 (2.8) | 20.2 (2.1) | .67 |
| Education level greater than high school, n (%) | 39 (60) | 32 (55) | .72 |
| Homosexual or bisexual or uncertain, n (%) | 55 (85) | 42 (72) | .15 |
| Married, n (%) | 9 (14) | 10 (17) | .79 |
| Family monthly income ≥ 7000 Yuan (US $1043.7), n (%) | 26 (40) | 26 (45) | .72 |
| Duration of HIV infection, median (IQR) | 1.42 (0.33 to 4.00) | 2.17 (0.55 to 4.21) | .61 |
| CES-D score, mean (SD) | 23.1 (6.6) | 23.9 (6.7) | .51 |
| WHOQOL-HIV BREF score, mean (SD) | 78.1 (9.5) | 77.1 (8.0) | .53 |
| PSS score, mean (SD) | 19.8 (4.2) | 20.1 (3.7) | .66 |
| SWCQ positive coping score, mean (SD) | 18.1 (5.8) | 17.7 (6.3) | .67 |
| SWCQ negative coping score, mean (SD) | 12.2 (3.6) | 11.4 (3.6) | .28 |
| GSES score, mean (SD) | 25.0 (5.5) | 23.8 (5.2) | .23 |
| HIV Stigma Scale score, mean (SD) | 37.6 (7.2) | 37.8 (5.8) | .82 |
| PHQ-9 score, mean (SD) | 9.8 (4.3) | 10.2 (4.1) | .54 |
| METs, median (IQR) | 840 (0 to 3840) | 620 (0 to 1800) | .32 |
| Abbreviations: SD, standard deviation; IQR, interquartile range; BMI, Body Mass Index; CES-D, the Center for Epidemiological Studies-Depression Scale; QOL, quality of life; GSES, General Self-efficacy Scale; PSS, Perceived Stress Scale; SWCQ, Simplified Ways of Coping Questionnaire; PHQ-9, 9-item Patient Health Questionnaire; METs, Metabolic equivalents. | | | |

**Table S5. Effects of the Run4Love Intervention on the Primary and Secondary Outcomes Without Missing Data Imputation**

| **Follow-up time** | **Run4Love intervention group** | | | |  | **Usual care group** | | | | **Between-group difference for mean change from baseline, mean difference (95% *CI*)** | ***P* value** |
| --- | --- | --- | --- | --- | --- | --- | --- | --- | --- | --- | --- |
|  | **n** | **Baseline,**  **mean (*SD*)** | **Follow-up,**  **mean difference (*SD*)** | **Within-group changes, mean difference (95% *CI*)** ^c^ |  | **n** | **Baseline, mean (*SD*)** | **Follow-up,**  **mean (*SD*)** | **Within-group changes, mean difference (95% *CI*)** ^c^ |  |  |
| CES-D^a^ |  |  |  |  |  |  |  |  |  |  |  |
| 3-month follow-up | 139 | 24.2 (6.4) | 17.9 (9.4) | −6.29 (−7.76 to −4.83) |  | 135 | 24.1 (6.8) | 23.9 (10.1) | −0.20 (−1.71 to 1.31) | −6.09 (−8.19 to −4.00) | <.001 |
| 6-month follow-up | 132 | 24.2 (6.5) | 17.6 (10.1) | −6.64 (−8.25 to −5.02) |  | 133 | 24.1 (6.7) | 24.1 (11.4) | −0.02 (−1.70 to 1.67) | −6.62 (−8.94 to −4.30) | <.001 |
| 9-month follow-up | 133 | 23.9 (6.3) | 17.9 (10.7) | −6.06 (−7.91 to −4.21) |  | 127 | 24.4 (7.0) | 23.4 (11.5) | −0.93 (−2.67 to 0.81) | −5.13 (−7.66 to −2.60) | <.001 |
| 1-year follow-up | 74 | 23.8 (5.9) | 18.2 (11.5) | −5.61 (−8.40 to −2.82) |  | 75 | 24.7 (7.7) | 24.7 (13.2) | 0.04 (−2.27 to 2.65) | −5.65 (−9.43 to −1.86) | .004 |
| 3-year follow-up | 85 | 24.5 (6.2) | 20.7 (11.1) | −3.85 (−6.18 to −1.51) |  | 92 | 24.5 (7.0) | 25.4 (13.1) | 0.99 (−1.40 to 3.37) | −4.84 (−8.16 to −1.51) | .005 |
| QOL^b^ |  |  |  |  |  |  |  |  |  |  |  |
| 3-month follow-up | 139 | 77.3 (9.2) | 82.5 (12.0) | 5.23 (3.62 to 6.83) |  | 135 | 76.3 (9.0) | 76.6 (11.1) | 0.37 (−0.95 to 1.68) | 4.86 (2.79 to 6.93) | <.001 |
| 6-month follow-up | 132 | 77.3 (9.2) | 83.5 (12.9) | 6.19 (4.43 to 7.94) |  | 133 | 76.7 (9.6) | 76.3 (13.0) | −0.39 (−1.93 to 1.16) | 6.57 (4.24 to 8.90) | <.001 |
| 9-month follow-up | 133 | 77.6 (9.3) | 83.5 (13.2) | 5.87 (3.78 to 7.96) |  | 127 | 76.1 (9.7) | 76.5 (13.3) | 0.49 (−1.28 to 2.26) | 5.38 (2.65 to 8.11) | <.001 |
| 1-year follow-up | 74 | 77.5 (8.3) | 84.3 (15.0) | 6.77 (3.58 to 9.97) |  | 75 | 75.3 (10.4) | 76.2 (15.6) | 0.85 (−1.71 to 3.42) | 5.92 (1.86 to 9.98) | .005 |
| 3-year follow-up | 85 | 76.9 (8.6) | 78.6 (13.1) | 1.74 (−0.68 to 4.17) |  | 92 | 76.3 (10.2) | 73.1 (14.8) | −3.14 (−5.62 to −0.65) | 4.88 (1.42 to 8.33) | .006 |
| Perceived stress PSS^a^ |  |  |  |  |  |  |  |  |  |  |  |
| 3-month follow-up | 139 | 20.0 (4.5) | 15.7 (5.8) | −4.32 (−5.18 to −3.47) |  | 135 | 21.0 (4.3) | 19.0 (5.4) | −1.93 (−2.76 to −1.10) | −2.39 (−3.58 to −1.20) | <.001 |
| 6-month follow-up | 132 | 20.0 (4.6) | 16.4 (5.9) | −3.52 (−4.44 to −2.59) |  | 133 | 20.9 (4.4) | 19.4 (6.1) | −1.53 (−2.4 to −0.65) | −1.99 (−3.26 to −0.72) | .002 |
| 9-month follow-up | 133 | 20.1 (4.6) | 16.1 (6.0) | −3.99 (−4.91 to −3.07) |  | 127 | 20.9 (4.3) | 18.7 (6.0) | −2.22 (−3.15 to −1.29) | −1.77 (−3.08 to −0.47) | .008 |
| 1-year follow-up | 74 | 19.8 (4.4) | 15.9 (6.4) | −3.91 (−5.23 to −2.51) |  | 75 | 21.4 (4.8) | 19.7 (7.2) | −1.69 (−3.15 to −0.24) | −2.21 (−4.21 to −0.21) | .03 |
| 3-year follow-up | 85 | 20.1 (4.6) | 18.0 (5.9) | −2.12 (−3.33 to −0.90) |  | 92 | 21.1 (4.8) | 20.3 (6.8) | −0.81 (−2.07 to 0.44) | 0.88 (−3.04 to 0.44) | .14 |
| SWCQ positive coping^b^ |  |  |  |  |  |  |  |  |  |  |  |
| 3-month follow-up | 139 | 18.4 (5.6) | 20.8 (7.3) | 2.42 (1.23 to 3.62) |  | 135 | 18.3 (5.9) | 17.7 (5.9) | −0.62 (−1.55 to 0.30) | 3.05 (1.54 to 4.55) | <.001 |
| 6-month follow-up | 132 | 18.6 (5.6) | 21.0 (7.5) | 2.45 (1.25 to 3.66) |  | 133 | 18.5 (6.1) | 17.4 (6.6) | −1.13 (−2.22 to −0.03) | 3.58 (1.96 to 5.20) | <.001 |
| 9-month follow-up | 133 | 18.5 (5.6) | 21.0 (7.8) | 2.43 (1.12 to 3.74) |  | 127 | 18.5 (6.2) | 18.3 (6.4) | −0.22 (−1.36 to 0.92) | 2.65 (0.92 to 4.38) | .003 |
| 1-year follow-up | 74 | 18.1 (4.7) | 21.6 (7.7) | 3.45 (1.58 to 5.31) |  | 75 | 18.1 (6.3) | 18.0 (7.0) | −0.09 (−1.43 to 1.24) | 3.54 (1.27 to 5.81) | .002 |
| 3-year follow-up | 85 | 18.6 (5.2) | 19.1 (6.9) | 0.54 (−1.14 to 2.22) |  | 92 | 18.7 (6.1) | 17.7 (6.3) | −1.02 (−2.30 to 0.26) | 1.56 (−0.53 to 3.64) | .14 |

**Table S5. Effects of the Run4Love Intervention on the Primary and Secondary Outcomes Without Missing Data Imputation (Continued)**

| **Follow-up time** | **Run4Love intervention group** | | | |  | **Usual care group** | | | | **Between-group difference for mean change from baseline, mean difference (95% *CI*)** | ***P* value** |
| --- | --- | --- | --- | --- | --- | --- | --- | --- | --- | --- | --- |
|  | **n** | **Baseline,**  **mean (*SD*)** | **Follow-up,**  **mean difference (*SD*)** | **Within-group changes, mean difference (95% *CI*)** ^c^ |  | **n** | **Baseline, mean (*SD*)** | **Follow-up,**  **mean (*SD*)** | **Within-group changes, mean difference (95% *CI*)** ^c^ |  |  |
| SWCQ negative coping^a^ |  |  |  |  |  |  |  |  |  |  |  |
| 3-month follow-up | 139 | 11.8 (3.8) | 11.1 (4.3) | −0.68 (−1.43 to 0.06) |  | 135 | 11.8 (3.9) | 11.4 (3.7) | −0.39 (−1.03 to 0.24) | −0.29 (−1.26 to 0.68) | .56 |
| 6-month follow-up | 132 | 11.9 (3.9) | 11.3 (4.4) | −0.55 (−1.27 to 0.18) |  | 133 | 11.7 (3.9) | 11.3 (4.1) | −0.38 (−1.08 to 0.33) | −0.17 (−1.17 to 0.83) | .74 |
| 9-month follow-up | 133 | 11.9 (3.9) | 11.7 (4.1) | −0.15 (−0.91 to 0.61) |  | 127 | 11.9 (4.0) | 11.9 (4.1) | −0.02 (−0.83 to 0.79) | −0.13 (−1.23 to 0.98) | .82 |
| 1-year follow-up | 74 | 11.3 (3.9) | 11.7 (4.0) | 0.47 (−0.55 to 1.49) |  | 75 | 12.1 (4.1) | 12.3 (4.7) | 0.23 (−0.81 to 1.26) | 0.25 (−1.19 to 1.69) | .74 |
| 3-year follow-up | 85 | 11.5 (4.0) | 12.1 (4.0) | 0.55 (−0.48 to 1.58) |  | 92 | 11.9 (4.0) | 11.8 (3.9) | −0.13 (−1.01 to 0.82) | 0.68 (−0.71 to 2.08) | .33 |
| Self-efficacy GSES^b^ |  |  |  |  |  |  |  |  |  |  |  |
| 3-month follow-up | 139 | 24.3 (5.2) | 26.6 (6.0) | 2.32 (1.41 to 3.24) |  | 135 | 23.2 (5.6) | 23.4 (5.8) | 0.18 (−0.68 to 1.03) | 2.15 (0.90 to 3.39) | <.001 |
| 6-month follow-up | 132 | 24.3 (5.3) | 26.4 (6.0) | 2.09 (1.10 to 3.08) |  | 133 | 23.4 (5.6) | 23.7 (5.7) | 0.29 (−0.67 to 1.26) | 1.80 (0.42 to 3.17) | .01 |
| 9-month follow-up | 133 | 24.4 (5.3) | 26.6 (5.8) | 2.18 (1.27 to 3.09) |  | 127 | 23.4 (5.7) | 23.8 (5.9) | 0.39 (−0.68 to 1.47) | 1.79 (0.38 to 3.19) | .01 |
| 1-year follow-up | 74 | 23.6 (5.2) | 26. 9(7.1) | 3.31 (1.78 to 4.84) |  | 75 | 22.3 (5.8) | 22.8 (6.4) | 0.51(−0.93 to 1.95) | 2.80 (0.72 to 4.89) | .009 |
| 3-year follow-up | 85 | 24.0 (4.9) | 25.1 (6.4) | 1.11 (−0.20 to 2.42) |  | 92 | 23.0 (5.9) | 22.7 (6.7) | −0.36 (−1.74 to 1.03) | 1.46 (0.96 to 3.37) | .13 |
| HIV Stigma Scale^a^ |  |  |  |  |  |  |  |  |  |  |  |
| 3-month follow-up | 139 | 37.3 (7.7) | 34.3 (9.2) | −3.04 (−4.29 to −1.78) |  | 135 | 38.0 (7.5) | 37.5 (8.3) | −0.50 (−1.59 to 0.58) | −2.53 (−4.19 to −0.88) | .003 |
| 6-month follow-up | 132 | 37.4 (7.9) | 34.3 (8.5) | −3.11 (−4.35 to −1.87) |  | 133 | 38.2 (7.5) | 37.4 (9.9) | −0.83 (−2.20 to 0.53) | −2.27 (−4.11 to −0.44) | .02 |
| 9-month follow-up | 133 | 37.0 (8.0) | 34.0 (9.0) | −3.05 (−4.36 to −1.75) |  | 127 | 38.1 (7.8) | 37.8 (10.0) | −0.27 (−1.61 to 1.08) | −2.78 (−4.65 to −0.92) | .004 |
| 1-year follow-up | 74 | 36.5 (7.7) | 32.6 (9.8) | −3.92 (−5.69 to −2.15) |  | 75 | 37.5 (9.0) | 37.6 (11.0) | 0.05 (−1.94 to 2.04) | −3.97 (−6.61 to −1.33) | .003 |
| 3-year follow-up | 85 | 36.7 (8.5) | 35.5 (9.7) | −1.22 (−2.89 to 0.44) |  | 92 | 38.1 (8.5) | 38.8 (10.3) | 0.75 (−1.07 to 2.57) | −1.97 (−4.42 to 0.48) | .12 |
| Depression severity PHQ-9^a^ | |  |  |  |  |  |  |  |  |  |  |
| 3-month follow-up | 139 | 10.2 (4.6) | 6.8 (4.1) | −3.43 (−4.19 to −2.68) |  | 135 | 10.8 (5.0) | 9.0 (4.6) | −1.81 (−2.59 to −1.02) | −1.62 (−2.71 to −0.54) | .003 |
| 6-month follow-up | 132 | 10.3 (4.6) | 8.5 (5.0) | −1.80 (−2.68 to −0.92) |  | 133 | 10.8 (5.2) | 11.2 (5.6) | 0.40 (−0.44 to 1.24) | −2.19 (−3.40 to −0.98) | <.001 |
| 9-month follow-up | 133 | 10.2 (4.7) | 9.2 (5.1) | −1.06 (−2.09 to −0.03) |  | 127 | 10.8 (5.2) | 10.9 (5.5) | 0.10 (−0.78 to 0.98) | −1.16 (−2.51 to 0.19) | .09 |
| 1-year follow-up | 74 | 10.0 (4.1) | 7.2 (5.3) | −2.80 (−4.08 to −1.65) |  | 75 | 10.8 (5.7) | 9.10 (5.9) | −1.69 (−2.92 to −0.47) | −1.17 (−2.89 to 0.54) | .18 |
| 3-year follow-up | 85 | 10.5 (4.7) | 8.5 (5.4) | −2.01 (−3.24 to −0.78) |  | 92 | 11.0 (5.6) | 9.22 (6.2) | −1.80 (−3.09 to −0.52) | −0.21 (−1.98 to 1.56) | .82 |

**Table S5. Effects of the Run4Love Intervention on the Primary and Secondary Outcomes Without Missing Data Imputation (Continued)**

| **Follow-up time** | **Run4Love intervention group** | | | |  | **Usual care group** | | | | **Between-group difference for mean change from baseline, mean difference (95% *CI*)** | ***P* value** |
| --- | --- | --- | --- | --- | --- | --- | --- | --- | --- | --- | --- |
|  | **n** | **Baseline,**  **mean (*SD*)** | **Follow-up,**  **mean difference (*SD*)** | **Within-group changes, mean difference (95% *CI*)** ^c^ |  | **n** | **Baseline, mean (*SD*)** | **Follow-up,**  **mean (*SD*)** | **Within-group changes, mean difference (95% *CI*)** ^c^ |  |  |
| Physical activity METs^b^ |  |  |  |  |  |  |  |  |  |  |  |
| 3-month follow-up | 139 | 3323 (6346) | 3052 (5822) | −270 (−1378 to 837) |  | 135 | 2855 (6354) | 4545 (16437) | 1689 (−579 to 3959) | −1960 (−4479 to 557) | 0.13 |
| 6-month follow-up | 132 | 3386 (6465) | 4405 (12003) | 1019 (−1019 to 3057) |  | 133 | 2870 (6408) | 3986 (9040) | 1115 (−768 to 3000) | −96 (−2859 to 2666) | 0.95 |
| 9-month follow-up | 133 | 3409 (6456) | 4734 (10363) | 1324 (−472 to 3121) |  | 127 | 2896 (6530) | 4602 (9243) | 1706 (−164 to 3576) | −381 (−2963 to 2200) | 0.77 |
| 1-year follow-up | 74 | 2910 (4941) | 2910 (4941) | −725 (−2795 to 1344) |  | 75 | 9752 (51996) | 9752(51996) | 7453 (−4596 to 19504) | −8,178 (−20394 to 4036) | 0.19 |
| 3-year follow-up | 85 | 2478 (4445) | 2478 (4445) | −480 (−1844 to 844) |  | 92 | 3183 (7537) | 9183(7537) | −49 (−1797 to 1699) | −431 (−2634 to 1772) | 0.70 |
| Abbreviations: SD, standard deviation; IQR, interquartile range; CI, confidence interval; CES-D, the Center for Epidemiological Studies-Depression Scale (higher scores indicate greater depression); QOL, Quality of life; GSES, General Self-efficacy Scale; PSS, Perceived Stress Scale; SWCQ, Simplified Ways of Coping Questionnaire; PHQ-9, 9-item Patient Health Questionnaire (higher scores indicate greater depression); METs, Metabolic equivalents.  ^a^ A higher score indicates a worse outcome.  ^b^ A higher score indicates a better outcome.  ^c^ Within-group changes are mean changes. | | | | | | | | | | | |

**Table S6. Effects of the Run4Love Intervention on the Primary and Secondary Outcomes (Full Results)**

| **Follow-up time** | **Run4Love intervention group (n=150)** | | |  | **Usual care group (n=150)** | | | **Between-group difference for mean change from baseline, mean difference (95% *CI*)** | ***P* value** |
| --- | --- | --- | --- | --- | --- | --- | --- | --- | --- |
|  | **Baseline, mean (*SD*)** | **Follow-up,**  **mean (*SD*)** | **Within-group changes, mean difference (95% *CI*)** ^c^ |  | **Baseline,**  **mean (*SD*)** | **Follow-up,**  **mean (*SD*)** | **Within-group changes,**  **Mean difference (95% *CI*)** ^c^ |  |  |
| CES-D^a^ |  |  |  |  |  |  |  |  |  |
| 3-month follow-up | 23.9 (6.4) | 17.7 (9.5) | −6.21 (−7.66 to −4.76) |  | 24.3 (6.9) | 23.8 (10.0) | −0.44 (−1.92 to 1.03) | −5.77 (−7.82 to −3.71) | <.001 |
| 6-month follow-up | 23.9 (6.4) | 17.6 (10.0) | −6.37 (−7.96 to −4.79) |  | 24.3 (6.9) | 24.0 (11.2) | −0.29 (−1.93 to 1.34) | −6.08 (−8.33 to −3.83) | <.001 |
| 9-month follow-up | 23.9 (6.4) | 17.8 (10.6) | −6.17 (−7.99 to −4.35) |  | 24.3 (6.9) | 23.4 (11.2) | −0.87 (−2.54 to 0.81) | −5.30 (−7.77 to −2.83) | <.001 |
| 1-year follow-up | 23.9 (6.4) | 18.1 (11.3) | −5.79 (−7.99 to −3.59) |  | 24.3 (6.9) | 23.3 (12.4) | −0.99 (−3.27 to 1.29) | −4.79 (−7.78 to −1.81) | .002 |
| 3-year follow-up | 23.9 (6.4) | 20.5 (11.2) | −3.47 (−5.64 to −1.31) |  | 24.3 (6.9) | 24.4 (12.7) | 0.15 (−2.07 to 2.38) | −3.63 (−6.71 to −0.54) | .02 |
| QOL^b^ |  |  |  |  |  |  |  |  |  |
| 3-month follow-up | 77.4 (9.0) | 82.6 (12.0) | 5.16 (3.55 to 6.76) |  | 76.6 (9.4) | 77.0 (11.3) | 0.36 (−0.96 to 1.68) | 4.79 (2.72 to 6.87) | <.001 |
| 6-month follow-up | 77.4 (9.0) | 83.7 (12.8) | 6.26 (4.50 to 8.01) |  | 76.6 (9.4) | 76.3 (12.7) | −0.34 (−1.87 to 1.19) | 6.60 (4.27 to 8.92) | <.001 |
| 9-month follow-up | 77.4 (9.0) | 83.3 (13.0) | 5.91 (3.89 to 7.93) |  | 76.6 (9.4) | 76.7 (13.1) | 0.07 (−1.66 to 1.80) | 5.84 (3.18 to 8.51) | <.001 |
| 1-year follow-up | 77.4 (9.0) | 82.8 (14.1) | 5.42 (2.88 to 7.95) |  | 76.6 (9.4) | 77.9 (14.7) | 1.26 (−1.08 to 3.60) | 4.15 (0.78 to 7.53) | .02 |
| 3-year follow-up | 77.4 (9.0) | 78.6 (13.7) | 1.13 (−1.23 to 3.48) |  | 76.6 (9.4) | 74.1 (14.3) | −2.5 (−4.96 to −0.05) | 3.63 (0.31 to 6.95) | .03 |
| Perceived stress PSS^a^ |  |  |  |  |  |  |  |  |  |
| 3-month follow-up | 20.0 (4.4) | 15.7 (5.7) | −4.23 (−5.08 to −3.38) |  | 20.7 (4.4) | 18.9 (5.5) | −1.78 (−2.61 to −0.95) | −2.45 (−3.63 to −1.27) | <.001 |
| 6-month follow-up | 20.0 (4.4) | 16.6 (5.8) | −3.35 (−4.23 to −2.46) |  | 20.7 (4.4) | 19.2 (6.0) | −1.46 (−2.32 to −0.60) | −1.88 (−3.10 to −0.67) | .003 |
| 9-month follow-up | 20.0 (4.4) | 16.1 (6.0) | −3.84 (−4.74 to −2.93) |  | 20.7 (4.4) | 18.7 (5.9) | −2.04 (−2.94 to −1.14) | −1.79 (−3.06 to −0.53) | .006 |
| 1-year follow-up | 20.0 (4.4) | 16.6 (6.14) | −3.38 (−4.70 to −2.06) |  | 20.7 (4.4) | 19.3 (6.6) | −1.41 (−2.74 to −0.08) | −1.97 (−3.68 to −0.25) | .03 |
| 3-year follow-up | 20.0 (4.4) | 18.0 (5.85) | −1.93 (−3.04 to −0.81) |  | 20.7 (4.4) | 19.9 (6.6) | −0.8 (−1.99 to 0.38) | −1.12 (−2.74 to 0.50) | .17 |
| SWCQ positive coping^b^ |  |  |  |  |  |  |  |  |  |
| 3-month follow-up | 18.4 (5.5) | 20.7 (7.3) | 2.35 (1.18 to 3.52) |  | 18.3 (6.2) | 17.8 (6.0) | −0.56 (−1.53 to 0.41) | 2.91 (1.39 to 4.43) | <.001 |
| 6-month follow-up | 18.4 (5.5) | 20.9 (7.4) | 2.48 (1.30 to 3.65) |  | 18.3 (6.2) | 17.4 (6.6) | −0.93 (−2.04 to 0.17) | 3.41 (1.80 to 5.02) | <.001 |
| 9-month follow-up | 18.4 (5.5) | 20.8 (7.7) | 2.44 (1.16 to 3.72) |  | 18.3 (6.2) | 18.2 (6.4) | −0.09 (−1.22 to 1.03) | 2.53 (0.85 to 4.21) | .003 |
| 1-year follow-up | 18.4 (5.5) | 21.0 (7.6) | 2.59 (0.95 to 4.23) |  | 18.3 (6.2) | 18.2 (7.1) | −0.16 (−1.59 to 1.27) | 2.75 (0.75 to 4.76) | .007 |
| 3-year follow-up | 18.4 (5.5) | 19.2 (6.9) | 0.78 (−0.84 to 2.40) |  | 18.3 (6.2) | 17.8 (6.4) | −0.53 (−1.84 to 0.79) | 1.31 (−0.74 to 3.35) | .21 |

**Table S6. Effects of the Run4Love Intervention on the Primary and Secondary Outcomes (Full Results, Continued)**

| **Follow-up time** | **Run4Love intervention group (n=150)** | | |  | **Usual care group (n=150)** | | | **Between-group difference for mean change from baseline, mean difference (95% *CI*)** | ***P* value** |
| --- | --- | --- | --- | --- | --- | --- | --- | --- | --- |
|  | **Baseline, mean (*SD*)** | **Follow-up,**  **mean (*SD*)** | **Within-group changes, mean difference (95% *CI*)** ^c^ |  | **Baseline,**  **mean (*SD*)** | **Follow-up,**  **mean (*SD*)** | **Within-group changes,**  **Mean difference (95% *CI*)** ^c^ |  |  |
| SWCQ negative coping^a^ |  |  |  |  |  |  |  |  |  |
| 3-month follow-up | 11.8 (3.8) | 11.1 (4.2) | −0.67 (−1.40 to 0.06) |  | 11.7 (3.9) | 11.5 (3.7) | −0.29 (−0.93 to 0.35) | −0.38 (−1.34 to 0.58) | .44 |
| 6-month follow-up | 11.8 (3.8) | 11.3 (4.4) | −0.45 (−1.17 to 0.27) |  | 11.7 (3.9) | 11.3 (4.1) | −0.44 (−1.14 to 0.26) | −0.01 (−1.00 to 0.99) | .99 |
| 9-month follow-up | 11.8 (3.8) | 11.7 (4.1) | −0.06 (−0.8 to 0.68) |  | 11.7 (3.9) | 11.8 (4.1) | 0.05 (−0.72 to 0.82) | −0.11 (−1.18 to 0.96) | .84 |
| 1-year follow-up | 11.8 (3.8) | 11.9 (4.3) | 0.11 (−0.81 to 1.03) |  | 11.7 (3.9) | 11.9 (4.6) | 0.19 (−0.76 to 1.15) | −0.08 (−1.39 to 1.22) | .90 |
| 3-year follow-up | 11.8 (3.8) | 12.1 (4.0) | 0.29 (−0.67 to 1.25) |  | 11.7 (3.9) | 11.6 (4.0) | −0.11 (−1.01 to 0.79) | 0.40 (−0.92 to 1.71) | .55 |
| Self-efficacy GSES^b^ |  |  |  |  |  |  |  |  |  |
| 3-month follow-up | 24.4 (5.2) | 26.6 (6.0) | 2.24 (1.33 to 3.15) |  | 23.3 (5.6) | 23.4 (5.8) | 0.08 (−0.77 to 0.94) | 2.16 (0.92 to 3.40) | <.001 |
| 6-month follow-up | 24.4 (5.2) | 26.5 (6.0) | 2.06 (1.09 to 3.03) |  | 23.3 (5.6) | 23.5 (5.7) | 0.20 (−0.76 to 1.15) | 1.86 (0.50 to 3.22) | .007 |
| 9-month follow-up | 24.4 (5.2) | 26.4 (5.9) | 2.01 (1.08 to 2.95) |  | 23.3 (5.6) | 23.8 (5.9) | 0.46 (−0.55 to 1.47) | 1.55 (0.19 to 2.91) | .03 |
| 1-year-follow-up | 24.4 (5.2) | 26.6 (6.7) | 2.22 (0.82 to 3.62) |  | 23.3 (5.6) | 23.5 (6.4) | 0.20 (−1.12 to 1.51) | 2.02 (0.18 to 3.87) | .03 |
| 3-year-follow-up | 24.4 (5.2) | 25.1 (6.4) | 0.73 (−0.52 to 1.98) |  | 23.3 (5.6) | 22.9 (6.5) | −0.42 (−1.72 to 0.87) | 1.15 (−0.62 to 2.93) | .20 |
| HIV Stigma Scale^a^ |  |  |  |  |  |  |  |  |  |
| 3-month follow-up | 37.1 (7.7) | 34.3 (9.1) | −2.85 (−4.09 to −1.61) |  | 38.0 (7.5) | 37.4 (8.3) | −0.56 (−1.65 to 0.53) | −2.29 (−3.93 to −0.65) | .006 |
| 6-month follow-up | 37.1 (7.7) | 34.2 (8.5) | −2.88 (−4.10 to −1.66) |  | 38.0 (7.5) | 37.2 (9.8) | −0.82 (−2.14 to 0.49) | −2.05 (−3.83 to −0.28) | .02 |
| 9-month follow-up | 37.1 (7.7) | 34.0 (9.0) | −3.15 (−4.43 to −1.86) |  | 38.0 (7.5) | 37.7 (9.9) | −0.28 (−1.60 to 1.04) | −2.87 (−4.71 to −1.03) | .002 |
| 1-year follow-up | 37.1 (7.7) | 33.5 (9.6) | −3.62 (−5.43 to −1.82) |  | 38.0 (7.5) | 36.9 (10.4) | −1.10 (−2.95 to 0.76) | −2.53 (−5.17 to 0.11) | .06 |
| 3-year follow-up | 37.1 (7.7) | 35.4 (9.9) | −1.70 (−3.43 to 0.03) |  | 38.0 (7.5) | 37.6 (10.1) | −0.43 (−2.14 to 1.28) | −1.27 (−3.69 to 1.14) | .30 |
| Depression severity PHQ-9^a^ |  |  |  |  |  |  |  |  |  |
| 3-month follow-up | 10.2 (4.5) | 6.8 (4.1) | −3.38 (−4.13 to −2.62) |  | 10.7 (5.1) | 8.9 (4.7) | −1.81 (−2.58 to −1.05) | −1.56 (−2.63 to −0.50) | .004 |
| 6-month follow-up | 10.2 (4.5) | 8.5 (4.9) | −1.68 (−2.54 to −0.81) |  | 10.7 (5.1) | 11.1 (5.5) | 0.34 (−0.49 to 1.16) | −2.01 (−3.20 to −0.83) | <.001 |
| 9-month follow-up | 10.2 (4.5) | 9.1 (5.1) | −1.01 (−1.99 to −0.03) |  | 10.7 (5.1) | 10.9 (5.4) | 0.16 (−0.70 to 1.02) | −1.17 (−2.46 to 0.13) | .08 |
| 1-year follow-up | 10.2 (4.5) | 7.2 (5.1) | −2.98 (−4.09 to −1.87) |  | 10.7 (5.1) | 8.5 (5.5) | −2.23 (−3.30 to −1.15) | −0.75 (−2.22 to 0.72) | .32 |
| 3-year follow-up | 10.2 (4.5) | 8.4 (5.4) | −1.75 (−2.87 to −0.63) |  | 10.7 (5.1) | 8.7 (5.9) | −2.06 (−3.16 to −0.97) | 0.31 (−1.24 to 1.87) | .69 |

**Table S6. Effects of the Run4Love Intervention on the Primary and Secondary Outcomes (Full Results, Continued)**

| **Follow-up time** | **Run4Love intervention group (n=150)** | | |  | **Usual care group (n=150)** | | | **Between-group difference for mean change from baseline, mean difference(95% *CI*)** | ***P* value** |
| --- | --- | --- | --- | --- | --- | --- | --- | --- | --- |
|  | **Baseline, mean (*SD*)** | **Follow-up,**  **mean (*SD*)** | **Within-group changes, mean difference (95% *CI*)** ^c^ |  | **Baseline,**  **mean (*SD*)** | **Follow-up,**  **mean (*SD*)** | **Within-group changes,**  **Mean difference (95% *CI*)** ^c^ |  |  |
| Physical activity METs^b^ |  |  |  |  |  |  |  |  |  |
| 3-month follow-up | 3225 (6189) | 3070 (6036) | −155 (−1301 to 990) |  | 2675 (6064) | 4418 (15755) | 1743 (−370 to 3856) | −1898 (−4285 to 489) | .12 |
| 6-month follow-up | 3225 (6189) | 4418 (11870) | 1193 (−775 to 3161) |  | 2675 (6064) | 3970 (9090) | 1296 (−525 to 3116) | −103 (−2769 to 2564) | .94 |
| 9-month follow-up | 3225 (6189) | 4707 (10136) | 1482 (−235 to 3199) |  | 2675 (6064) | 4466 (8959) | 1792 (76 to 3508) | −310 (−2713 to 2094) | .80 |
| 1-year follow-up | 3225 (6189) | 4193(20017) | 969 (−3414 to 5352) |  | 2675 (6064) | 6850 (37316) | 4176 (−2041 to 10393) | −3206 (−10734 to 4320) | .40 |
| 3-year follow-up | 3225 (6189) | 2788 (5470) | −436 (−1814 to 941) |  | 2675 (6064) | 2933 (6860) | 258 (−1142 to 1660) | −695 (−2674 to 1283) | .49 |
| Abbreviations: SD, standard deviation; IQR, interquartile range; CI, confidence interval; CES-D, the Center for Epidemiological Studies-Depression Scale (higher scores indicate greater depression); QOL, Quality of life; GSES, General Self-efficacy Scale; PSS, Perceived Stress Scale; SWCQ, Simplified Ways of Coping Questionnaire; PHQ-9, 9-item Patient Health Questionnaire (higher scores indicate greater depression); METs, Metabolic equivalents.  ^a^ A higher score indicates a worse outcome.  ^b^ A higher score indicates a better outcome.  ^c^ Within-group changes are mean changes. | | | | | | | | | |

**Table S7. GEE Results of the Primary Outcome and Secondary Outcomes^a^ (Full Results)**

| **Variables** | **Beta coefficient** | **Standard error** | **95% *CI*** | ***P* value** |
| --- | --- | --- | --- | --- |
| **CES-D** |  |  |  |  |
| Intercept | 32.45 | 5.67 | 21.29 to 43.62 | <.001 |
| Group | −0.02 | 0.78 | −1.55 to 1.52 | .98 |
| 3-month vs BL | −0.46 | 0.75 | −1.93 to 1.01 | .54 |
| 6-month vs BL | −0.28 | 0.84 | −1.92 to 1.36 | .74 |
| 9-month vs BL | −0.82 | 0.85 | −2.49 to 0.85 | .34 |
| 1-year vs BL | −0.99 | 1.15 | −3.24 to 1.26 | .39 |
| 3-year vs BL | 0.15 | 1.12 | −2.04 to 2.35 | .89 |
| Group×(3-month vs BL) | −5.67 | 1.04 | −7.72 to −3.62 | <.001 |
| Group×(6-month vs BL) | −6.06 | 1.15 | −8.31 to −3.81 | <.001 |
| Group×(9-month vs BL) | −5.29 | 1.26 | −7.76 to −2.82 | <.001 |
| Group×(1-year vs BL) | −4.79 | 1.51 | −7.76 to −1.83 | .002 |
| Group×(3-year vs BL) | −3.63 | 1.56 | −6.69 to −0.56 | .02 |
| **QOL** |  |  |  |  |
| Intercept | 68.11 | 6.94 | 54.47 to 81.75 | <.001 |
| Group | 0.18 | 1.05 | −1.87 to 2.24 | .86 |
| 3-month vs BL | 0.36 | 0.66 | −0.94 to 1.66 | .59 |
| 6-month vs BL | −0.26 | 0.76 | −1.76 to 1.23 | .73 |
| 9-month vs BL | 0.10 | 0.86 | −1.60 to 1.79 | .91 |
| 1-year vs BL | 1.26 | 1.18 | −1.05 to 3.57 | .28 |
| 3-year vs BL | −2.50 | 1.23 | −4.93 to −0.08 | .04 |
| Group×(3-month vs BL) | 4.63 | 1.04 | 2.60 to 6.67 | <.001 |
| Group×(6-month vs BL) | 6.28 | 1.17 | 3.98 to 8.57 | <.001 |
| Group×(9-month vs BL) | 5.64 | 1.32 | 3.05 to 8.24 | <.001 |
| Group×(1-year vs BL) | 4.15 | 1.70 | 0.81 to 7.50 | .02 |
| Group×(3-year vs BL) | 3.63 | 1.68 | 0.34 to 6.92 | .03 |
| **Perceived stress PSS** |  |  |  |  |
| Intercept | 25.25 | 2.68 | 19.98 to 30.52 | <.001 |
| Group | −0.63 | 0.50 | −1.60 to 0.35 | .21 |
| 3-month vs BL | −1.79 | 0.42 | −2.60 to −0.97 | <.001 |
| 6-month vs BL | −1.48 | 0.43 | −2.33 to 0.63 | <.001 |
| 9-month vs BL | −2.07 | 0.45 | −2.96 to −1.17 | <.001 |
| 1-year vs BL | −1.41 | 0.66 | −2.72 to −0.10 | .03 |
| 3-year vs BL | −0.80 | 0.59 | −1.97 to 0.36 | .18 |
| Group×(3-month vs BL) | −2.45 | 0.60 | −3.62 to −1.28 | <.001 |
| Group×(6-month vs BL) | −1.90 | 0.62 | −3.13 to −0.68 | .002 |
| Group×(9-month vs BL) | −1.74 | 0.66 | −3.03 to −0.46 | .008 |
| Group×(1-year vs BL) | −1.97 | 0.86 | −3.67 to −0.26 | .02 |
| Group×(3-year vs BL) | −1.12 | 0.82 | −2.73 to 0.48 | .17 |

**Table S7. GEE Results of the Primary Outcome and Secondary Outcomes ^a^ (Full Results, Continued)**

| **Variables** | **Beta coefficient** | **Standard error** | **95% *CI*** | ***P* value** |
| --- | --- | --- | --- | --- |
| **SWCQ positive coping** |  |  |  |  |
| Intercept | 14.91 | 3.15 | 8.72 to 21.09 | <.001 |
| Group | −0.26 | 0.66 | −1.55 to 1.03 | .69 |
| 3-month vs BL | −0.56 | 0.49 | −1.52 to 0.39 | .25 |
| 6-month vs BL | −0.99 | 0.56 | −2.09 to 0.12 | .08 |
| 9-month vs BL | −0.20 | 0.57 | −1.32 to 0.93 | .73 |
| 1-year vs BL | −0.16 | 0.71 | −1.57 to 1.25 | .82 |
| 3-year vs BL | −0.53 | 0.66 | −1.83 to 0.77 | .42 |
| Group×(3-month vs BL) | 2.90 | 0.76 | 1.40 to 4.40 | <.001 |
| Group×(6-month vs BL) | 3.40 | 0.82 | 1.79 to 5.02 | <.001 |
| Group×(9-month vs BL) | 2.58 | 0.85 | 0.90 to 4.27 | .003 |
| Group×(1-year vs BL) | 2.75 | 1.01 | 0.76 to 4.74 | .007 |
| Group×(3-year vs BL) | 1.31 | 1.03 | −0.72 to 3.34 | .21 |
| **SWCQ negative coping** |  |  |  |  |
| Intercept | 12.56 | 1.79 | 9.05 to 16.07 | <.001 |
| Group | 0.03 | 0.44 | −0.83 to 0.89 | .95 |
| 3-month vs BL | −0.30 | 0.32 | −0.93 to 0.33 | .35 |
| 6-month vs BL | −0.40 | 0.35 | −1.09 to 0.28 | .25 |
| 9-month vs BL | 0.03 | 0.38 | −0.72 to 0.78 | .94 |
| 1-year vs BL | 0.19 | 0.48 | −0.75 to 1.14 | .69 |
| 3-year vs BL | −0.11 | 0.45 | −1.00 to 0.78 | .81 |
| Group×(3-month vs BL) | −0.35 | 0.49 | −1.31 to 0.61 | .47 |
| Group×(6-month vs BL) | −0.001 | 0.50 | −0.97 to 0.97 | >.99 |
| Group×(9-month vs BL) | −0.11 | 0.53 | −1.16 to 0.94 | .84 |
| Group×(1-year vs BL) | −0.08 | 0.66 | −1.38 to 1.21 | .90 |
| Group×(3-year vs BL) | 0.40 | 0.66 | −0.91 to 1.70 | .55 |
| **Self-efficacy GSES** |  |  |  |  |
| Intercept | 22.12 | 3.05 | 16.12 to 28.11 | <.001 |
| Group | 0.92 | 0.62 | −0.30 to 2.13 | .14 |
| 3-month vs BL | 0.08 | 0.43 | −0.77 to 0.92 | .86 |
| 6-month vs BL | 0.20 | 0.48 | −0.74 to 1.14 | .68 |
| 9-month vs BL | 0.42 | 0.52 | −0.60 to 1.44 | .42 |
| 12-month vs BL | 0.20 | 0.66 | −1.10 to 1.49 | .77 |
| 36-month vs BL | −0.42 | 0.65 | −1.70 to 0.85 | .52 |
| Group×(3-month vs BL) | 2.12 | 0.63 | 0.89 to 3.35 | <.001 |
| Group×(6-month vs BL) | 1.76 | 0.69 | 0.41 to 3.11 | .01 |
| Group×(9-month vs BL) | 1.54 | 0.70 | 0.17 to 2.91 | .02 |
| Group×(12-month vs BL) | 2.02 | 0.93 | 0.19 to 3.85 | .03 |
| Group×(36-month vs BL) | 1.15 | 0.90 | −0.61 to 2.92 | .20 |

**Table S7. GEE Results of the Primary Outcome and Secondary Outcomes ^a^ (Full Results, Continued)**

| **Variables** | **Beta coefficient** | **Standard error** | **95% *CI*** | ***P* value** |
| --- | --- | --- | --- | --- |
| **HIV Stigma Scale** |  |  |  |  |
| Intercept | 41.54 | 5.18 | 31.36 to 51.73 | <.001 |
| Group | −0.87 | 0.88 | −2.60 to 0.86 | .33 |
| 3-month vs BL | −0.59 | 0.56 | −1.68 to 0.51 | .29 |
| 6-month vs BL | −0.83 | 0.67 | −2.14 to 0.48 | .21 |
| 9-month vs BL | −0.29 | 0.66 | −1.59 to 1.00 | .66 |
| 1-year vs BL | −1.10 | 0.93 | −2.92 to 0.73 | .24 |
| 3-year vs BL | −0.43 | 0.86 | −2.12 to 1.26 | .62 |
| Group×(3-month vs BL) | −2.32 | 0.83 | −3.95 to −0.69 | .005 |
| Group×(6-month vs BL) | −2.12 | 0.90 | −3.88 to −0.36 | .02 |
| Group×(9-month vs BL) | −2.88 | 0.92 | −4.69 to −1.07 | .002 |
| Group×(1-year vs BL) | −2.53 | 1.33 | −5.14 to 0.09 | .06 |
| Group×(3-year vs BL) | −1.27 | 1.22 | −3.67 to 1.12 | .30 |
| **Depression severity PHQ-9** |  |  |  |  |
| Intercept | 14.86 | 2.75 | 9.46 to 20.28 | <.001 |
| Group | −0.45 | 0.55 | −1.53 to 0.63 | .41 |
| 3-month vs BL | −1.82 | 0.39 | −2.58 to −1.06 | <.001 |
| 6-month vs BL | −1.70 | 0.41 | −2.49 to −0.90 | <.001 |
| 9-month vs BL | −1.91 | 0.42 | −2.74 to −1.08 | <.001 |
| 1-year vs BL | −2.23 | 0.54 | −3.33 to −1.17 | <.001 |
| 3-year vs BL | −2.06 | 0.55 | −3.14 to −0.98 | <.001 |
| Group×(3-month vs BL) | −1.55 | 0.54 | −2.60 to −0.49 | .004 |
| Group×(6-month vs BL) | −1.73 | 0.58 | −2.88 to −0.59 | .003 |
| Group×(9-month vs BL) | −0.98 | 0.64 | −2.23 to 0.28 | .13 |
| Group×(1-year vs BL) | −0.75 | 0.74 | −2.21 to 0.71 | .31 |
| Group×(3-year vs BL) | 0.31 | 0.78 | −1.23 to 1.85 | .69 |

**Table S7. GEE Results of the Primary Outcome and Secondary Outcomes ^a^ (Full Results, Continued)**

| **Variables** | **Beta coefficient** | **Standard error** | **95% *CI*** | ***P* value** |
| --- | --- | --- | --- | --- |
| **Physical activity METs** |  |  |  |  |
| Intercept | −3588.14 | 6158.32 | −15694.56 to 8518.29 | .56 |
| Group | 695.76 | 716.06 | −708.67 to 2100.18 | .33 |
| 3-month vs BL | 1686.91 | 1051.80 | −376.02 to 3749.84 | .11 |
| 6-month vs BL | 1274.83 | 902.23 | −494.93 to 3044.58 | .16 |
| 9-month vs BL | 1805.31 | 872.47 | 93.88 to 3516.73 | .04 |
| 1-year vs BL | 4176.08 | 3135.08 | −1972.94 to 10325.1 | .18 |
| 3-year vs BL | 258.78 | 704.80 | −1125.24 to 1642.8 | .71 |
| Group×(3-month vs BL) | −1874.75 | 1195.47 | −4219.53 to 470.02 | .12 |
| Group×(6-month vs BL) | −67.00 | 1351.83 | −2718.83 to 2584.83 | .96 |
| Group×(9-month vs BL) | −314.61 | 1226.13 | −2719.70 to 2090.47 | .80 |
| Group×(1-year vs BL) | −3206.96 | 3809.88 | −10682.89 to 4268.97 | .40 |
| Group×(3-year vs BL) | −695.36 | 999.28 | −2659.52 to 1268.8 | .49 |
| Abbreviations: CI, confidence interval; BL, Baseline; BMI, Body Mass Index; CES-D, the Center for Epidemiological Studies-Depression Scale; QOL, quality of life; PSS, Perceived Stress Scale; GSES, General Self-efficacy Scale; SWCQ, Simplified Ways of Coping Questionnaire; PHQ-9, 9-item Patient Health Questionnaire; METs, Metabolic equivalents.  ^a^ Adjusted for age, gender, BMI, education, sexual orientation, family monthly income, marital status, duration of HIV infection, and employment.  Explanation of the model: Intercept, mean value in usual care group at baseline; Group, between-group difference at baseline; 3-, 6-, 9-,12-, 36-month vs BL, difference from baseline in usual care group at 3, 6, 9, 12 or 36 months; Group×(3-, 6-, 9-, 12-, 36-month vs BL), interaction effect, representing between-group difference for mean change from baseline. | | | | |

**Table S8. GEE Results of the Primary Outcome and Secondary Outcomes ^a^ Without Missing Data Imputation**

| **Variables** | **Beta coefficient** | **Standard error** | **95% *CI*** | ***P* value** |
| --- | --- | --- | --- | --- |
| **CES-D** |  |  |  |  |
| Intercept | 34.12 | 4.44 | 25.40to 42.80 | <.001 |
| Group | 0.03 | 0.78 | −1.49 to 1.56 | .97 |
| 3-month vs BL | −0.41 | 0.74 | −1.86 to 1.05 | .58 |
| 6-month vs BL | −0.27 | 0.84 | −1.91 to 1.37 | .75 |
| 9-month vs BL | −0.90 | 0.84 | −2.56 to 0.75 | .28 |
| 1-year vs BL | −0.38 | 1.08 | −2.48 to 1.73 | .73 |
| 3-year vs BL | 0.51 | 1.12 | −1.69 to 2.71 | .65 |
| Group×(3-month vs BL) | −5.78 | 1.05 | −7.83 to −3.73 | <.001 |
| Group×(6-month vs BL) | −6.20 | 1.15 | −8.46 to −3.94 | <.001 |
| Group×(9-month vs BL) | −5.31 | 1.25 | −7.76 to −2.87 | <.001 |
| Group×(1-year vs BL) | −5.23 | 1.59 | −8.35 to −2.12 | .001 |
| Group×(3-year vs BL) | −4.07 | 1.52 | −7.05 to −1.09 | .008 |
| **QOL** |  |  |  |  |
| Intercept | 66.98 | 5.72 | 55.80 to78.20 | <.001 |
| Group | 0.15 | 1.04 | −1.89 to 2.19 | .89 |
| 3-month vs BL | 0.31 | 0.65 | −0.97 to 1.58 | .64 |
| 6-month vs BL | −0.27 | 0.77 | −1.77 to 1.24 | .73 |
| 9-month vs BL | 0.24 | 0.86 | −1.45 to 1.93 | .78 |
| 1-year vs BL | 0.77 | 1.10 | −1.38 to 2.93 | .48 |
| 3-year vs BL | −2.61 | 1.17 | −4.90 to −0.323 | .03 |
| Group×(3-month vs BL) | 4.70 | 1.05 | 2.65 to 6.75 | <.001 |
| Group×(6-month vs BL) | 6.42 | 1.18 | 4.11 to 8.73 | <.001 |
| Group×(9-month vs BL) | 5.64 | 1.35 | 2.99 to 8.28 | <.001 |
| Group×(1-year vs BL) | 4.83 | 1.75 | 1.40 to 8.26 | .006 |
| Group×(3-year vs BL) | 3.98 | 1.59 | 0.87 to 7.10 | .01 |
| **Perceived stress PSS** |  |  |  |  |
| Intercept | 26.60 | 2.25 | 22.20 to 31.00 | <.001 |
| Group | −0.58 | 0.50 | −1.56 to 0.40 | .25 |
| 3-month vs BL | −1.84 | 0.41 | −2.64 to −1.03 | <.001 |
| 6-month vs BL | −1.49 | 0.43 | −2.34 to −0.65 | <.001 |
| 9-month vs BL | −2.11 | 0.46 | −3.00 to −1.22 | <.001 |
| 1-year vs BL | −1.41 | 0.61 | −2.61 to −0.20 | .02 |
| 3-year vs BL | −0.75 | 0.59 | −1.90 to 0.40 | .20 |
| Group×(3-month vs BL) | −2.40 | 0.59 | −3.57 to −1.24 | <.001 |
| Group×(6-month vs BL) | −1.93 | 0.63 | −3.17 to −0.69 | .002 |
| Group×(9-month vs BL) | −1.78 | 0.64 | −3.05 to −0.52 | .006 |
| Group×(1-year vs BL) | −2.20 | 0.86 | −3.88 to −0.52 | .01 |
| Group×(3-year vs BL) | −1.34 | 0.80 | −2.91 to 0.23 | .09 |

**Table S8. GEE Results of the Primary Outcome and Secondary Outcomes ^a^ Without Missing Data Imputation (Continued)**

| **Variables** | **Beta coefficient** | **Standard error** | **95% *CI*** | ***P* value** |
| --- | --- | --- | --- | --- |
| **SWCQ positive coping** |  |  |  |  |
| Intercept | 15.60 | 2.82 | 10.00 to 21.10 | <.001 |
| Group | −0.29 | 0.65 | −1.57 to 0.99 | .66 |
| 3-month vs BL | −0.58 | 0.46 | −1.48 to 0.32 | .21 |
| 6-month vs BL | −0.99 | 0.55 | −2.06 to 0.08 | .07 |
| 9-month vs BL | −0.20 | 0.56 | −1.29 to 0.89 | .72 |
| 1-year vs BL | −0.27 | 0.60 | −1.45 to 0.91 | .66 |
| 3-year vs BL | 0.81 | 0.60 | −1.99 to 0.37 | .18 |
| Group×(3-month vs BL) | 2.91 | 0.76 | 1.43 to 4.39 | <.001 |
| Group×(6-month vs BL) | 3.49 | 0.80 | 1.92 to 5.07 | <.001 |
| Group×(9-month vs BL) | 2.62 | 0.85 | 0.96 to 4.29 | .002 |
| Group×(1-year vs BL) | 3.03 | 1.04 | 1.01 to 5.06 | .003 |
| Group×(3-year vs BL) | 1.50 | 0.98 | −0.43 to 3.43 | .13 |
| **SWCQ negative coping** |  |  |  |  |
| Intercept | 11.10 | 1.64 | 7.88 to 14.30 | <.001 |
| Group | 0.04 | 0.43 | −0.81 to 0.89 | .93 |
| 3-month vs BL | −0.36 | 0.31 | −0.96 to 0.25 | .25 |
| 6-month vs BL | −0.43 | 0.34 | −1.11 to 0.24 | .21 |
| 9-month vs BL | −0.06 | 0.39 | −0.82 to 0.69 | .87 |
| 1-year vs BL | 0.32 | 0.45 | −0.56 to 1.20 | .47 |
| 3-year vs BL | −0.13 | 0.44 | −0.98 to 0.73 | .77 |
| Group×(3-month vs BL) | −0.31 | 0.48 | −1.26 to 0.64 | .52 |
| Group×(6-month vs BL) | −0.01 | 0.50 | −0.98 to 0.96 | .98 |
| Group×(9-month vs BL) | −0.07 | 0.54 | −1.12 to 0.98 | .89 |
| Group×(1-year vs BL) | −0.20 | 0.63 | −1.44 to 1.03 | .75 |
| Group×(3-year vs BL) | 0.45 | 0.63 | −0.79 to 1.68 | .48 |
| **Self-efficacy GSES** |  |  |  |  |
| Intercept | 20.60 | 2.66 | 15.40 to 25.80 | <.001 |
| Group | 0.90 | 0.61 | −0.30 to 2.10 | .14 |
| 3-month vs BL | 0.09 | 0.42 | −0.73 to 0.90 | .83 |
| 6-month vs BL | 0.23 | 0.47 | −0.70 to 1.16 | .63 |
| 9-month vs BL | 0.40 | 0.52 | −0.62 to 1.42 | .44 |
| 1-year vs BL | 0.11 | 0.61 | −1.08 to 1.30 | .85 |
| 3-year vs BL | −0.52 | 0.62 | −1.75 to 0.70 | .40 |
| Group×(3-month vs BL) | 2.15 | 0.62 | 0.93 to 3.36 | <.001 |
| Group×(6-month vs BL) | 1.79 | 0.68 | 0.44 to 3.13 | .009 |
| Group×(9-month vs BL) | 1.67 | 0.69 | 0.32 to 3.03 | .02 |
| Group×(1-year vs BL) | 2.32 | 0.93 | 0.50 to 4.13 | .01 |
| Group×(3-year vs BL) | 1.22 | 0.88 | −0.50 to 2.95 | .16 |

**Table S8. GEE Results of the Primary Outcome and Secondary Outcomes ^a^ Without Missing Data Imputation (Continued)**

| **Variables** | **Beta coefficient** | **Standard error** | **95% *CI*** | ***P* value** |
| --- | --- | --- | --- | --- |
| **HIV Stigma Scale** |  |  |  |  |
| Intercept | 41.40 | 4.21 | 33.20 to 49.70 | <.001 |
| Group | −0.68 | 0.88 | −2.42 to 1.05 | .44 |
| 3-month vs BL | −0.61 | 0.54 | −1.67 to 0.45 | .26 |
| 6-month vs BL | −0.76 | 0.67 | −2.07 to 0.55 | .26 |
| 9-month vs BL | −0.25 | 0.66 | −1.54 to 1.04 | .70 |
| 1-year vs BL | −0.76 | 0.85 | −2.43 to −0.91 | .37 |
| 3-year vs BL | 0.11 | 0.82 | −1.50 to 1.73 | .89 |
| Group×(3-month vs BL) | −2.42 | 0.83 | −4.04 to −0.80 | .003 |
| Group×(6-month vs BL) | −2.36 | 0.90 | −4.13 to −0.59 | .009 |
| Group×(9-month vs BL) | −2.97 | 0.92 | −4.78 to −1.16 | .001 |
| Group×(1-year vs BL) | −2.89 | 1.15 | −5.15 to −0.64 | .01 |
| Group×(3-year vs BL) | −1.80 | 1.15 | −4.07 to 0.46 | .12 |
| **Depression severity PHQ-9** |  |  |  |  |
| Intercept | 15.20 | 2.33 | 10.60 to 19.70 | <.001 |
| Group | −0.43 | 0.56 | −1.52 to 0.66 | .44 |
| 3-month vs BL | −1.86 | 0.38 | −2.61 to −1.11 | <.001 |
| 6-month vs BL | −1.71 | 0.40 | −2.49 to −0.92 | <.001 |
| 9-month vs BL | −1.97 | 0.42 | −2.78 to −1.16 | <.001 |
| 1-year vs BL | −1.96 | 0.51 | −2.96 to−0.96 | <.001 |
| 3-year vs BL | −1.93 | 0.58 | −3.06 to −0.80 | <.001 |
| Group×(3-month vs BL) | −1.53 | 0.54 | −2.59 to −0.47 | .004 |
| Group×(6-month vs BL) | −1.80 | 0.58 | −2.94 to −0.65 | .002 |
| Group×(9-month vs BL) | −0.94 | 0.64 | −2.20 to 0.32 | .14 |
| Group×(1-year vs BL) | −0.94 | 0.75 | −2.41 to 0.52 | .21 |
| Group×(3-year vs BL) | 0.11 | 0.80 | −1.46 to 1.67 | .89 |

**Table S8. GEE Results of the Primary Outcome and Secondary Outcomes ^a^ Without Missing Data Imputation (Continued)**

| **Variables** | **Beta coefficient** | **Standard error** | **95% *CI*** | ***P* value** |
| --- | --- | --- | --- | --- |
| **Physical activity METs** |  |  |  |  |
| Intercept | −2195.1 | 3876.91 | −9793.7 to 5403.49 | 0.57 |
| Group | 716.75 | 694.55 | −644.54 to 2078.04 | 0.30 |
| 3-month vs BL | 1823.37 | 1171.19 | −472.12 to 4118.86 | 0.12 |
| 6-month vs BL | 1291.96 | 882.59 | −437.88 to 3021.8 | 0.14 |
| 9-month vs BL | 1833.39 | 901.31 | 66.86 to 3599.92 | 0.04 |
| 1-year vs BL | 7088.79 | 5944.59 | −4562.38 to 18739.97 | 0.23 |
| 3-year vs BL | 170.1 | 771.23 | −1341.48 to 1681.69 | 0.83 |
| Group×(3-month vs BL) | −2036.21 | 1288.5 | −4561.63 to 489.21 | 0.11 |
| Group×(6-month vs BL) | −111.12 | 1358.49 | −2773.71 to 2551.47 | 0.94 |
| Group×(9-month vs BL) | −321.52 | 1261.97 | −2794.93 to 2151.89 | 0.80 |
| Group×(1-year vs BL) | −7201.1 | 6011.46 | −18983.34 to 4581.15 | 0.23 |
| Group×(3-year vs BL) | −709.37 | 975.15 | −2620.63 to 1201.89 | 0.47 |
| Abbreviations: CI, confidence interval; BL, Baseline; BMI, Body Mass Index; CES-D, the Center for Epidemiological Studies-Depression Scale; QOL, quality of life; PSS, Perceived Stress Scale; SWCQ, Simplified Ways of Coping Questionnaire; GSES, General Self-efficacy Scale; PHQ-9, 9-item Patient Health Questionnaire; METs, Metabolic equivalents.  ^a^ Adjusted for age, gender, BMI, education, sexual orientation, family monthly income, marital status, duration of HIV infection, and employment. Explanation of the model: Intercept, mean value in usual care group at baseline; Group, between-group difference at baseline; 3-, 6-, 9-,12-, 36-month vs BL, difference from baseline in usual care group at 3, 6, 9 ,12 or 36 months; Group×(3-, 6-, 9-,12-, 36-month vs BL), interaction effect, representing between-group difference for mean change from baseline. | | | | |

**Figure S1. Secondary Outcomes Over Time for the Run4Love Intervention Group vs Control Group**


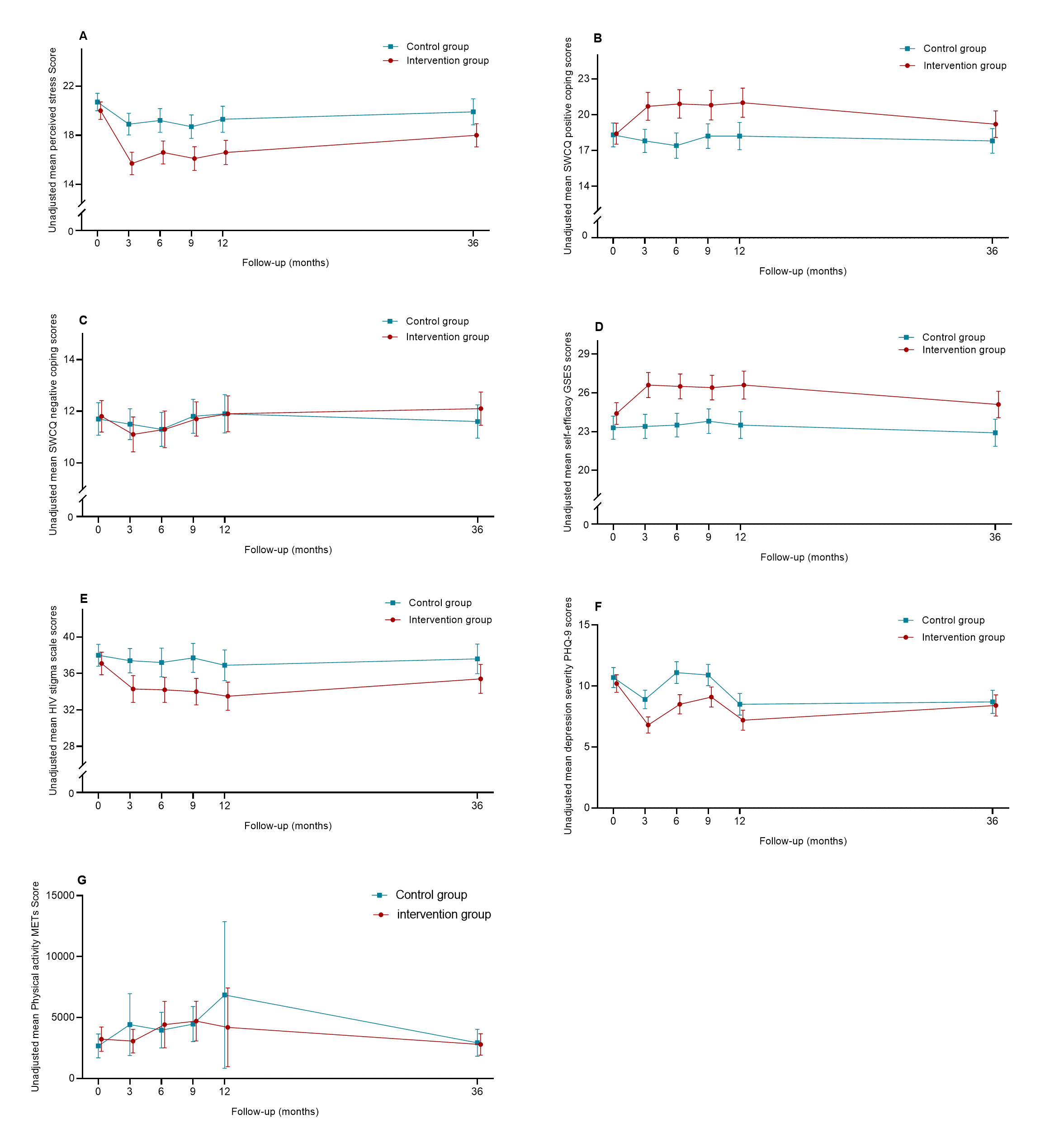


Abbreviations: PSS, Perceived Stress Scale; SWCQ, Simplified Ways of Coping Questionnaire; GSES, General Self-efficacy Scale; PHQ-9, 9-item Patient Health Questionnaire; METs, Metabolic equivalents.

Error bars indicate 95% CIs.
